# Systematic review with meta-analysis: real-world effectiveness and safety of vedolizumab in patients with inflammatory bowel disease

**DOI:** 10.1007/s00535-018-1480-0

**Published:** 2018-06-04

**Authors:** Stefan Schreiber, Axel Dignass, Laurent Peyrin-Biroulet, Greg Hather, Dirk Demuth, Mahmoud Mosli, Rebecca Curtis, Javaria Mona Khalid, Edward Vincent Loftus

**Affiliations:** 1 Department of Internal Medicine I and Institute of Clinical Molecular Biology, University-Hospital Schleswig-Holstein, Christian-Albrechts-University, Rosalind-Franklin-Strasse 12, 24105 Kiel, Germany; 2 Department of Medicine 1, Agaplesion Markus Hospital, Goethe University, Frankfurt, Germany; 3 Inserm U954 and Gastroenterology Department, Nancy University Hospital, Lorraine University, Vandoeuvre-lès-Nancy, France; 4 Takeda Oncology, Takeda Global Research and Development, Boston, MA USA; 5 Global Medical Affairs, Takeda International-UK Branch, London, UK; 6 Department of Medicine, King Abdulaziz University, Jeddah, Saudi Arabia; 7 Department of Gastroenterology and Hepatology, Mayo Clinic College of Medicine, Rochester, MN USA

**Keywords:** Vedolizumab, Inflammatory bowel disease, Ulcerative colitis, Crohn’s disease, Real-world effectiveness

## Abstract

**Background:**

Selective patient recruitment can produce discrepancies between clinical trial results and real-world effectiveness.

**Methods:**

A systematic literature review and meta-analysis were conducted to assess vedolizumab real-world effectiveness and safety in patients with ulcerative colitis (UC) or Crohn’s disease (CD). MEDLINE, MEDLINE In-Process, EMBASE, and Cochrane databases were searched for real-world studies of vedolizumab in adult patients with UC/CD reporting clinical response, remission, corticosteroid-free remission, UC/CD-related surgery or hospitalization, mucosal healing, or safety published from May 1, 2014–June 22, 2017. Response and remission rates were combined in random-effects meta-analyses.

**Results:**

At treatment week 14, 32% of UC patients [95% confidence interval (CI) 27–39%] and 30% of CD patients (95% CI 25–34%) were in remission; and at month 12, 46% for UC (95% CI 37–56%) and 30% for CD (95% CI 20–42%). For UC, the rates of corticosteroid-free remission were 26% at week 14 (95% CI 20–34%) and 42% at month 12 (95% CI 31–53%); for CD they were 25% at week 14 (95%, CI 20–31%) and 31% at month 12 (95%, CI 20–45%). At month 12, 33–77% of UC and 6–63% of CD patients had mucosal healing. Nine percent of patients reported serious adverse events.

**Conclusions:**

Vedolizumab demonstrated real-world effectiveness in patients with moderate-to-severely active UC or CD, with approximately one-half and one-third of patients, respectively, in remission at treatment month 12. These findings are consistent with clinical trial data and support the long-term benefit–risk profile of vedolizumab.

**Electronic supplementary material:**

The online version of this article (10.1007/s00535-018-1480-0) contains supplementary material, which is available to authorized users.

## Introduction

Current treatment options for inflammatory bowel diseases (IBD) include aminosalicylates, corticosteroids (CS), thiopurines, calcineurin inhibitors, anti-cytokines, and anti-integrins [1, 2]. Vedolizumab is a gut-selective, humanized, monoclonal antibody that binds to α_4_β_7_ integrins, selectively blocking gut-selective lymphocyte trafficking [3, 4]. Vedolizumab efficacy and safety in moderate-to-severely active ulcerative colitis (UC) and Crohn’s disease (CD) were established by the GEMINI clinical trials [5–7], with marketing approval granted in May 2014 in the USA and later in Europe [8, 9]. Clinical guidelines recommend vedolizumab for UC not previously treated with biologic therapy [10], and for UC or CD that is refractory to conventional or anti-tumor necrosis factor-alpha (TNFα) treatment [1, 2].

Strict inclusion criteria used in randomized controlled trials (RCTs) can limit the patient population and generalizability of trial results to clinical practice, with the latter further compromised by IBD patient heterogeneity [11, 12]. Indeed, up to two-thirds of patients with IBD might be ineligible to participate in RCTs of biologics [11, 13]. An additional hindrance is the increasing unwillingness of patients to accept placebo control. Randomized controlled trials are, therefore, unlikely to fully represent the real-world IBD population. However, physicians require real-world effectiveness data to complement clinical trial results and inform treatment decisions. Assessing the treatment quality and effect size in clinical practice and evaluating the strength of this evidence through systematic literature reviews and meta-analyses can provide such data. Summation can overcome potential bias associated with individual studies and address challenges associated with the transferability of RCT findings; systematic literature reviews are, therefore, at the top of the evidence hierarchy as defined by the Oxford Centre for Evidence-Based Medicine [14, 15].

Data from the GEMINI 3 trial suggest that the full effect of vedolizumab-induced clinical remission in patients with CD may not be apparent before treatment week 10 [7]. The European Summary of Product Characteristics and US Prescribing Information both recommend that vedolizumab treatment of UC and CD should be discontinued if a therapeutic benefit is not observed by week 14 (by week 10 in UC in Europe) [8, 9]. Real-world data allow evaluation of the optimal time points for assessing clinical effectiveness and when concomitant therapies should be adjusted based on therapeutic response outside of RCT protocol-defined assessments.

Given that vedolizumab is relatively new and the number of treated patients is increasing, ongoing safety monitoring is essential. Real-world data from large cohorts can further characterize a drug’s safety profile not fully elucidated in clinical trials [16, 17]. We sought to systematically review and summarize published literature on real-world effectiveness and safety of vedolizumab studies and conduct a meta-analysis of effectiveness data.

## Materials and methods

### Study selection

A systematic review of MEDLINE, MEDLINE In-Process, EMBASE and Cochrane (May 1, 2014–June 22, 2017), and searches of clinicaltrials.gov and the World Health Organization International Clinical Trials Registry Platform were completed. Conference proceedings from 2015 to June 2017 were searched. Two researchers reviewed relevant publications independently, with disagreements resolved by discussion or a third reviewer. Studies were eligible if they included real-world evidence (e.g., medical record review, database, registry) and an adult patient population (≥ 18 years when initiating vedolizumab) receiving vedolizumab (Takeda Pharmaceuticals International, Inc., Deerfield, IL) for IBD (UC, CD, or unspecified/indeterminate colitis) and if outcomes reported were of interest. English and non-English language studies were eligible for inclusion. Studies were excluded if the total patient population was < 10, if vedolizumab was used off-label, or if safety data were reported at event level only (no denominator). Investigators were contacted for unpublished data (unpublished clinical data provided courtesy of Dr. Mark A. Samaan and Dr. Peter Irving from their UK study, 2016) and conference abstracts, and manual backward citation tracking of references (including studies) were performed to identify additional relevant studies [18–22].

### Data extraction and outcome measures

One researcher used predefined parameters to extract all data using a piloted form and, after this, a second researcher performed data checks for accuracy. Information obtained for each eligible study included author, year of publication, geographic location, and clinical outcomes reported. Patient characteristics included disease duration, age, sex, prior medication history, and IBD-related surgeries. The primary outcome measure was clinical remission; secondary outcome measures were clinical response, CS-free clinical remission, mucosal healing, endoscopic improvement, surgery and hospitalization rates, dose-escalation rates, and safety. Clinical response, clinical remission, and CS-free clinical remission rates (classified according to summarized measures in Table S1) (Samaan and Irving, 2016) [13, 23–48] were collected at weeks 6, 14, 26–30 (month 6), and 46–54 (month 12), where available. Subanalyses were performed to determine clinical remission rates by geographic region and in patients who were anti-TNFα-naive.

### Grading of evidence

Studies were assessed using the Oxford Centre for Evidence-Based Medicine 2011 Levels of Evidence, which evaluates the strength of evidence (including quality and bias) based on study design [15, 49]. One reviewer appraised each study and assigned a level from 1 (high quality or low risk of bias) to 5 (low quality or high risk of bias) (Table S2) [15, 49], with uncertainty resolved by discussion with a second reviewer.

### Statistical analyses

Meta-analyses were performed to combine clinical response, clinical remission, and CS-free clinical remission rates using R statistical software (version 3.2.2; R Foundation; Vienna, Austria) with the “meta” package (version 4.3-2). When multiple publications were available for a study, data from the most recent cohort were used for the combined analyses. Weighted mean clinical response, clinical remission, and CS-free clinical remission rates and corresponding 95% confidence intervals (CIs) were calculated using the DerSimonian–Laird random-effects model to account for between-study heterogeneity [50]. Where mean/median values and 95% CIs were reported, data were used as stated (calculated using the binomial distribution if 95% CIs were not reported). For studies reporting safety, IBD-related surgery or hospitalization, and dose escalation, the proportion of events was calculated. For mucosal healing or endoscopic improvement, analyses were based on either a cumulative incidence approach or as a proportion of those receiving endoscopy.

Study heterogeneity was determined using the *I*^2^ statistic (which describes the variability in the effect estimate that results from heterogeneity rather than sampling error [51]) and the Q-statistic (*P* < 0.05 was considered significant and suggested statistical heterogeneity). When ≥ 10 studies reporting the same outcome were available, publication bias was assessed using Egger’s weighted regression statistic, with *P* < 0.05 suggesting a higher likelihood of bias [52].

## Results

### Study and patient characteristics

Of 1542 publications identified, 89 publications (*N* = 9486; *n* = 4532 CD; *n* = 3216 UC; *n* = 1738 IBD unspecified/indeterminate/other) were eligible to be included in this review (Figure S1). Eleven studies (*n* = 1692) did not report separate UC and CD rates [18, 20, 22, 53–60]. Six studies focused on CD, 5 focused on UC, and 61 examined both conditions. Eighteen studies were full-text articles and 73 were conference proceedings. Most studies [40] were conducted in the USA, followed by Europe [30]. The grading of quality of evidence of the studies (Table S2) [15, 49] ranged from 3 (12 publications) to 4 (77 publications; Table S3) (Samaan and Irving 2016) [13, 18, 20, 22–48, 53–110].

The meta-analysis included 21 studies reporting clinical response (*n* = 2310) and 23 reporting clinical remission rates (*n* = 2298) (18 studies included an analyses of both outcomes; Table S1) (Samaan and Irving, 2016) [13, 23–34, 36–48]. Ten studies reported CS-free clinical remission rates (Table S1) (Samaan and Irving, 2016) [13, 23, 24, 28, 29, 31, 36, 38, 42, 46] and 10 reported mucosal healing or endoscopic improvement (Fig. 5; Figure S2) [34, 40, 60, 69]. Of 46 studies reporting safety outcomes, most were for UC/CD combined, rather than by separate indication (Table S4) [13, 18, 20, 24, 26, 27, 31, 32, 37, 38, 40–42, 44–46, 53, 55, 56, 58, 59, 61, 64–68, 70–74, 78–80, 84, 89, 91–95, 97, 98, 102, 104–109].

Patient demographics are described in Table S3 (Samaan and Irving, 2016) [13, 18, 20, 22–48, 53–110]. The mean patient age was 40.9 years (range 34.3–67.1; 39 studies); the mean disease duration was 9.8 years (range 2.9–18; 22 studies); and the mean percentage of patients with prior anti-TNFα therapy was 80.4% (range 0–100%; 42 studies).

### Primary outcome

#### Clinical remission

**Fig. 1:**
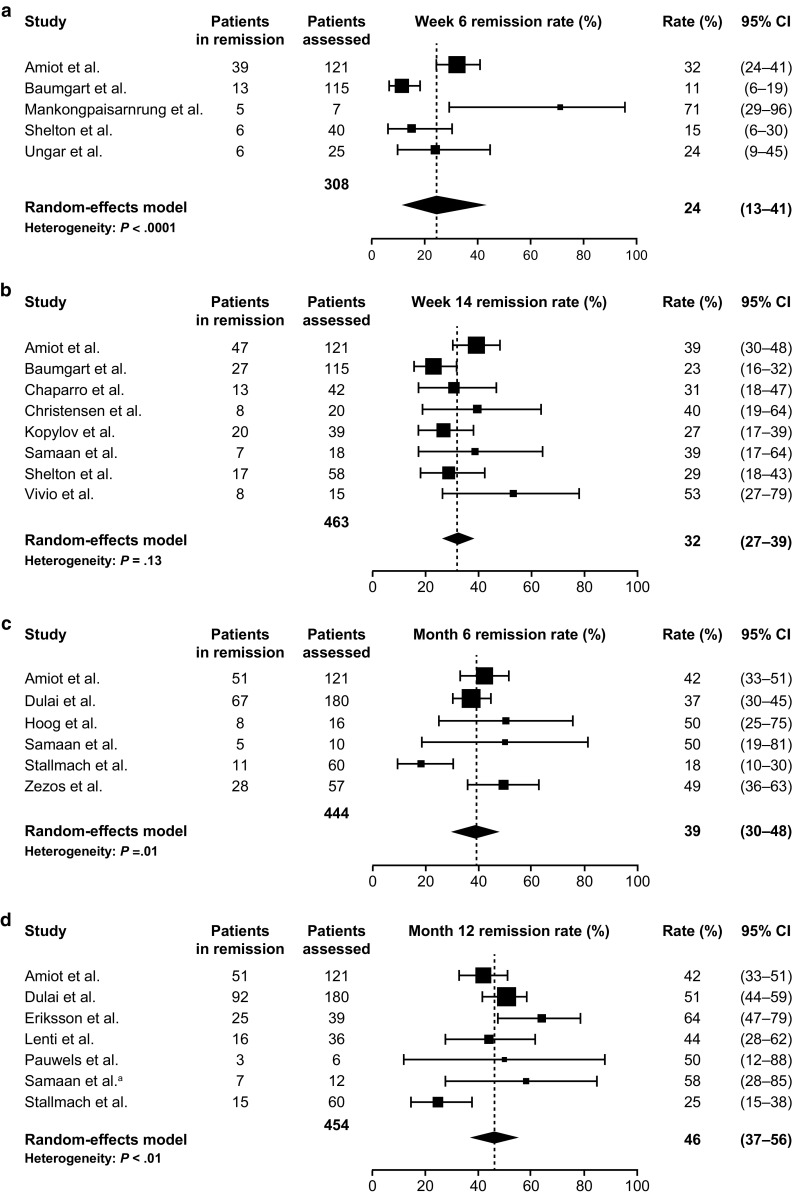
Meta-analysis of clinical remission rates among patients with ulcerative colitis receiving vedolizumab at the time points: **a** week 6; **b** week 14; **c** 6 months; and **d** 12 months. The size of each square represents the weight assigned to each study based on sample size. Error bars represent 95% CIs. Diamonds represent the point estimate of the averaged study rates; the lateral tips of the diamonds represent 95% CIs. *CI* confidence interval. Data from Amiot et al. [65], Baumgart et al. [24], Mankongpaisarnrung et al. [33], Shelton et al. [13], Ungar et al. [39], Chaparro et al. [25], Christensen et al. [27] Kopylov et al. [86], Samaan et al. [36], Vivio et al. [40], Dulai et al. [28], Hoog et al. [30], Stallmach et al. [38], Zezos et al. [42], Eriksson et al. [29], Lenti et al. [32], Pauwels et al. [34], Samaan et al.^a^. ^a^Unpublished clinical data provided courtesy of Dr. Mark A. Samaan and Dr. Peter Irving from their UK study, 2016

**Fig. 2:**
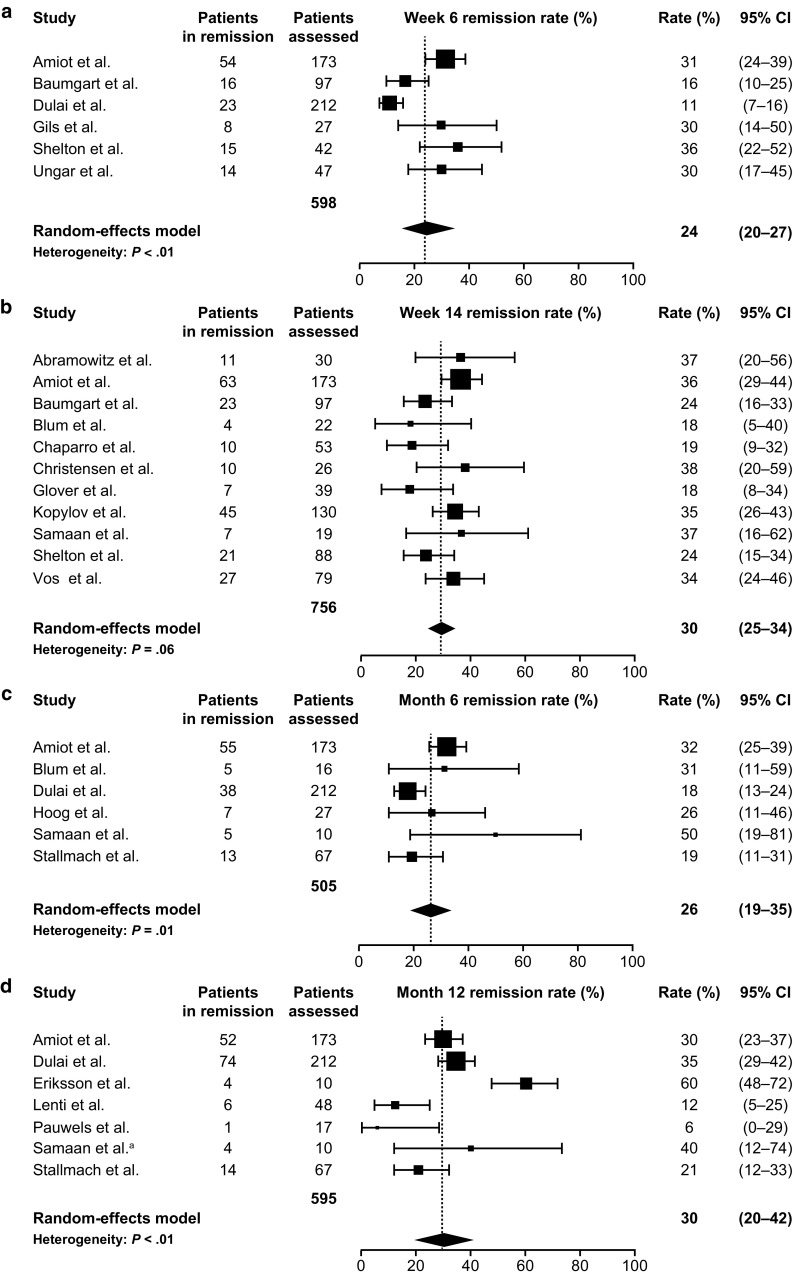
Meta-analysis of clinical remission rates among patients with Crohn’s disease receiving vedolizumab at the time points: **a** week 6; **b** week 14; **c** 6 months; and **d** 12 months. The size of each square represents the weight assigned to each study based on sample size. Error bars represent 95% CIs. Diamonds represent the point estimate of the averaged study rates; the lateral tips of the diamonds represent 95% CIs. *CI* confidence interval. Data from Amiot et al. [23], Baumgart et al. [24], Dulai et al. [46], Gils et al. [47], Shelton et al. [13], Ungar et al. [39], Abramowitz et al. [43], Blum et al. [44], Chaparro et al. [25], Christensen et al. [27], Glover et al. [48], Kopylov et al. [86], Samaan et al. [36], De Vos et al. [45], Hoog et al. [30], Stallmach et al. [38], Eriksson et al. [29], Lenti et al. [32], Pauwels et al. [34], Samaan et al.^a^. ^a^Unpublished clinical data provided courtesy of Dr. Mark A. Samaan and Dr. Peter Irving from their UK study, 2016

Clinical remission was assessed in 18 studies in UC, 18 in CD, and 13 in both populations (Table S1) (Samaan and Irving, 2016) [13, 23–25, 27–34, 36, 38–40, 42–48]. In UC, clinical remission was achieved in 24% of patients at week 6 (95% CI 13–41%) and 32% at week 14 (95% CI 27–39%), which increased to 39% at 6 months (95% CI 30–48%) and 46% at 12 months (95% CI 37–56%) (Fig. 1) (Samaan and Irving, 2016) [13, 24, 25, 27–30, 32–34, 36, 38–40, 42, 65, 86]. In CD, clinical remission was achieved in 24% of patients at week 6 (95% CI 20–27%), 30% at week 14 (95% CI 25–34%), 26% at 6 months (95% CI 19–35%), and 30% at 12 months (95% CI 20–42%) (Fig. 2) (Samaan and Irving, 2016) [13, 23–25, 27, 29, 30, 32, 34, 36, 38, 39, 43–48, 86].

Between-study heterogeneity was evident for all groups included in UC and CD remission analyses (*I*^2^ = 38–84%). However, Egger’s weighted regression for CD at week 14 indicated no publication bias (Egger’s *P* = 0.16).

### Secondary outcomes

#### Clinical response

Clinical response was evaluated in 16 studies in UC (Samaan and Irving, 2016) [13, 23–32, 34, 36–38, 41] and 20 in CD [23–27, 29–32, 34, 36–38, 41, 43–47] (Table S1) (Samaan and Irving, 2016) [13, 23–38, 41, 43–47]. Combined clinical response rates in UC were 43% at week 6 (95% CI 38–49%), 56% at week 14 (95% CI 50–62%), and 52% at 12 months (95% CI 37–65%) (Table S5). In CD, the combined clinical response rate was 56% at week 6 (95% CI 46–65%), 58% at week 14 (95% CI 51–64%), and 40% at 12 months (95% CI 29–52%). Ulcerative colitis studies showed low to moderate between-study heterogeneity, except for those included in the 12-month response rate analysis (*I*^2^ = 85%; *P* < 0.001) and week 6 remission rate analysis (*I*^2^ = 82%; *P* < 0.01). For CD, between-study heterogeneity was evident for all analyses (*P* < 0.01 for all), with an* I*^2^ of 43–84%, suggesting moderate to high between-study heterogeneity.

#### Corticosteroid-free clinical remission

**Fig. 3:**
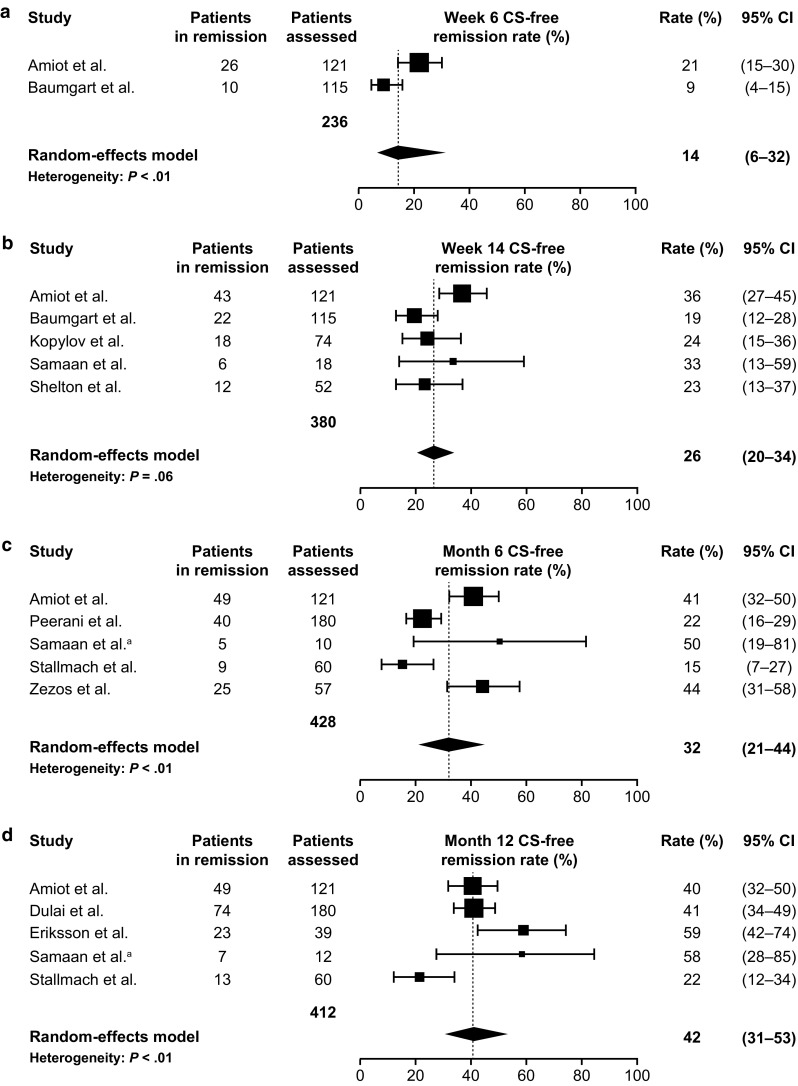
Meta-analysis of CS-free clinical remission rates among patients with ulcerative colitis receiving vedolizumab at the time points: **a** week 6; **b** week 14; **c** 6 months; and **d** 12 months. The size of each square represents the weight assigned to each study based on sample size. Error bars represent 95% CIs. Diamonds represent the point estimate of the averaged study rates; the lateral tips of the diamonds represent 95% CIs. *CI* confidence interval, *CS* corticosteroid. Data from Amiot et al. [65], Baumgart et al. [24], Kopylov et al. [86], Samaan et al. [36], Shelton et al. [13], Peerani et al. [35], Samaan et al.^a^, Stallmach et al. [38], Zezos et al. [42], Dulai et al. [28], Eriksson et al. [29]. ^a^Unpublished clinical data provided courtesy of Dr. Mark A. Samaan and Dr. Peter Irving from their UK study, 2016

Corticosteroid-free clinical remission was assessed in 9 studies in UC (Samaan and Irving, 2016) [13, 23, 24, 28, 29, 31, 36, 38, 42] and 8 in CD (Samaan and Irving, 2016) [13, 23, 24, 29, 31, 36, 38, 46] (8 reported both UC and CD CS-free clinical remission; Table S1). In patients with UC, CS-free clinical remission was achieved in 14% at week 6 (95% CI 6–32%), 26% at week 14 (95% CI 20–34%), and 32% at 6 months (95% CI 21–45%), with the rate increasing to 42% at 12 months (95% CI 31–53%) (Fig. 3) (Samaan and Irving, 2016) [13, 24, 28, 29, 35, 36, 38, 42, 65, 86].

**Fig. 4:**
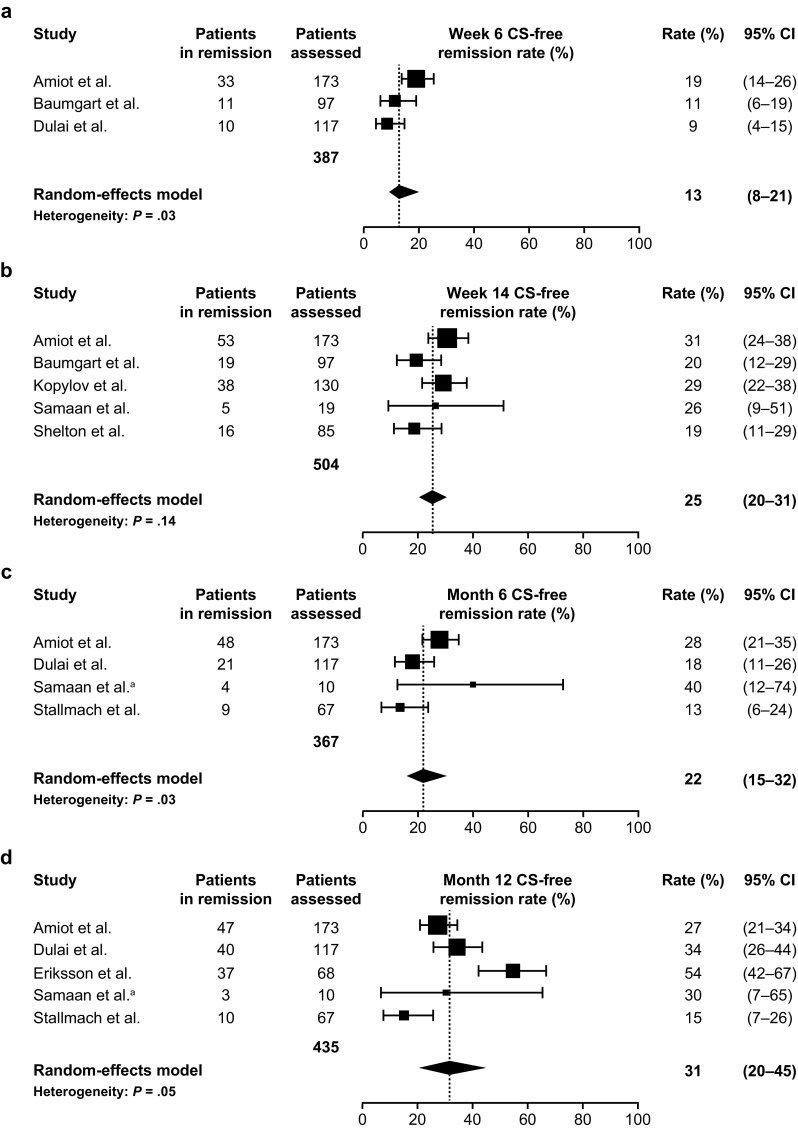
Meta-analysis of CS-free clinical remission rates among patients with Crohn’s disease receiving vedolizumab at the time points: **a** week 6; **b** week 14; **c** 6 months; and **d** 12 months. The size of each square represents the weight given to each study based on sample size. Error bars represent 95% CIs. Diamonds represent the point estimate of the averaged study rates; the lateral tips of the diamonds represent 95% CIs. *CI* confidence interval, *CS* corticosteroid. Data from Amiot et al. [23], Baumgart et al. [24], Dulai et al. [46], Kopylov et al. [86], Samaan et al. [36], Shelton et al. [13], Dulai et al. [46], Samaan et al.^a^, Stallmach et al. [38], Eriksson et al. [29]. ^a^Unpublished clinical data provided courtesy of Dr. Mark A. Samaan and Dr. Peter Irving from their UK study, 2016

In CD, CS-free clinical remission was achieved by 13% at week 6 (95% CI 8–21%), 25% at week 14 (95% CI 20–31%), and 22% at 6 months (95% CI 15–32%) and was maintained at 31% to 12 months (95% CI 20–45%) (Fig. 4) (Samaan and Irving, 2016) [13, 23, 24, 29, 36, 38, 46, 86]. Between-study heterogeneity was evident for all groups included in UC CS-free clinical remission analyses (*P* ≤ 0.03 for all) and for all groups in the CD CS-free clinical remission analyses, other than week 14 (*P* = 0.14). Corticosteroid-free response results are summarized in Table S5.

#### Mucosal healing and endoscopic improvement

**Fig. 5:**
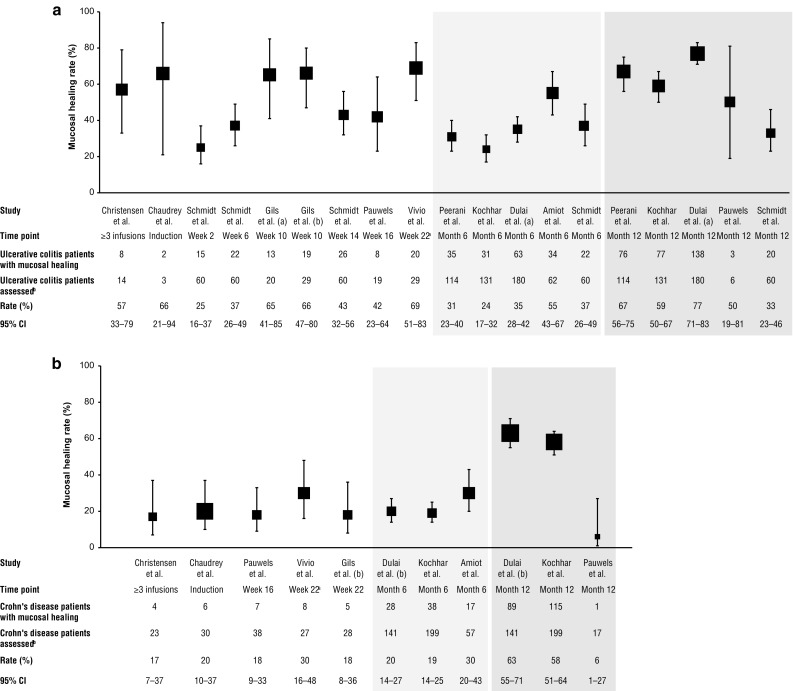
Mucosal healing rates among patients with ulcerative colitis (**a**) or Crohn’s disease (**b**) receiving vedolizumab. Square size represents the weight given to each study, based on sample size. Error bars represent 95% CIs. *CI* confidence interval. Christensen et al. [69], Chaudrey et al. [26], Schmidt et al. [103], Gils et al. (a) [47], Gils et al. (b) [77], Pauwels et al. [34], Vivio et al. [40], Peerani et al. [35], Kochhar et al. [83], Dulai et al. (a) [28], Amiot et al. [23], Dulai et al. (b) [46]. ^a^Median time point. ^b^Only patients with ≥ 1 follow-up assessment at the specified time point were included in the analyses. Data from the VICTORY Consortium, which contributed the majority of mucosal healing data, used a cumulative incidence analysis, and remaining studies employed a “complete” case approach

Twelve studies reported mucosal healing (Fig. 5) [23, 26, 28, 34, 35, 40, 46, 47, 69, 77, 83, 103], and 4 studies reported endoscopic improvement (Figure S2) [34, 40, 60, 69]. Mucosal healing rates ranged from 24 to 55% in patients with UC and 19–30% in patients with CD at month 6. At month 12, mucosal healing rates ranged from 33 to 77% in patients with UC and 6–63% in patients with CD. In a study of patients with UC or CD, endoscopic improvement was observed in 76 and 52% of patients, respectively, at a median time point of 22 weeks (Figure S2) [40]. In patients with CD, rates of endoscopic improvement were consistent over time with 53 and 50% of patients experiencing an improvement at week 16 and week 52, respectively (Figure S2) [34].

#### IBD-related surgery and hospitalization rates

Three real-world IBD studies [62, 99, 110] included in our systematic review (Table S6) [13, 22, 23, 26, 31, 35, 36, 40–42, 44–46, 54–56, 62–64, 67, 69, 71, 74, 76, 80–82, 85–88, 90, 91, 93, 96–102, 110] demonstrated reductions in hospitalization rates in the post-treatment versus pre-treatment period.

#### Vedolizumab dose-escalation rates

Rates of vedolizumab dose escalation ranging from 4 to 60% up to week 54 were reported in 8 real-world studies (Table S7) [23, 33, 37, 41, 46, 57, 75, 108]. Dose-escalation rates were lower in biologic-naive (4–20%) versus biologic-experienced patients (6–29%) [57, 75]. Among 4 studies reporting dose-escalation outcomes, 31–81% of patients recaptured response (Table S7) [33, 37, 46, 108].

#### Vedolizumab safety

Safety outcomes were reported in 46 studies (Table S4) [13, 18, 20, 26, 27, 31, 32, 37, 38, 41, 42, 45, 46, 55, 56, 59, 61, 64, 65, 68, 70–74, 78–80, 84, 89, 94, 97, 102, 104, 105, 107, 109] over a vedolizumab exposure/follow-up period of 0.5–12 months (exposure/follow-up data available for 27 studies). Overall adverse event (AE) rates were reported in 23 studies (0–67% of patients; *n* = 2358) and infections in 12 studies (range 5–24%; *n* = 1176). Serious AEs (range 0–13%) were reported in 4 studies (*n* = 857), and serious infections (range 4–10%) were reported in 3 studies (*n* = 832). Postoperative AEs were reported in 4 studies (range 8–65%) and serious postoperative AEs in 1 study (43%). The most common AEs were upper respiratory tract infections including nasopharyngitis (range 1–21%), arthralgia (range < 1–20%), *Clostridium difficile* infection (range < 1–20%), and fatigue (range 1–19%). Infusion-related reactions were uncommon, as were flu/flu-like infections, pruritus, and paresthesia (≤ 7% for all).

### Subgroup analysis

#### Clinical remission rates by geographic location

**Fig. 6:**
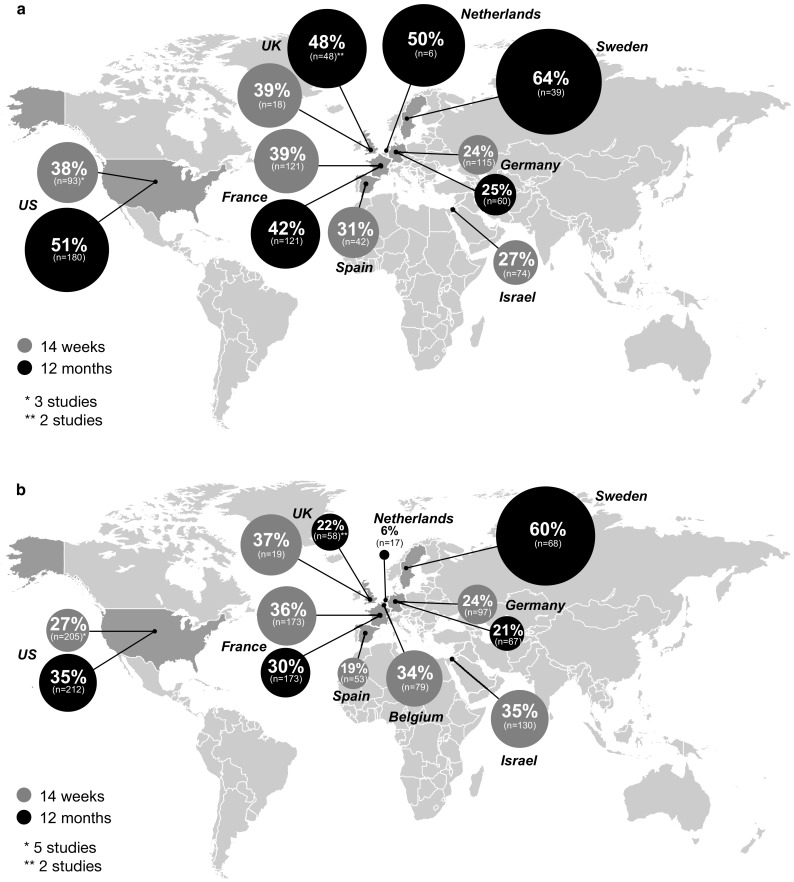
Subgroup analysis by geographical location showing clinical remission rates among patients with **a** ulcerative colitis and **b** Crohn’s disease. The size of each data bubble is proportional to the corresponding country clinical remission rates. Unless specified otherwise, one study was reported at each geographical location. Random-effects meta-analysis of single proportions was used to calculate an overall proportion in cases of > 1 study

A subgroup analysis by geographic location showed variable combined remission rates among patients with UC at week 14 [range 24% (Germany) to 39% (France and UK)] and month 12 [range 25% (Germany) to 64% (Sweden)] (Fig. 6a). Among studies conducted in the USA, remission rates were 38% (95% CI 25–52%) at week 14 and 51% at month 12. Remission rates among patients with CD also varied by geographic location (week 14: range, 19% [Spain] to 37% [UK]; month 12: range, 6% [Netherlands] to 60% [Sweden]) (Fig. 6b). Among studies conducted in the USA, remission rates were 27% (95% CI 20–35%) at week 14 and 35% at month 12.

#### Effectiveness in biologic-naive patients

In biologic-naive patients with UC, clinical remission was achieved in 51% of patients at week 14 (95% CI 40–62%) and 61% of patients at 12 months (95% CI 48–72%) (Figure S3) [24, 28, 31, 36, 38]. In biologic-naive patients with CD, clinical remission was achieved in 48% of patients at week 14 (95% CI 28–68%) and 44% of patients at 12 months (95% CI 18–75%) (Figure S4) [24, 31, 36, 38].

## Discussion

To the best of our knowledge, to date this is the most comprehensive meta-analysis of real-world clinical response and remission rates for vedolizumab over 12 months of treatment, incorporating data from both peer-reviewed full-text manuscripts and abstracts. Real-world effectiveness data provide valuable evidence to support the efficacy observed in RCTs, because trial patients may not be representative of the real-world IBD population [11].

In UC, clinical remission was achieved in approximately one-third of patients at 14 weeks and in approximately one-half of patients at 12 months. In CD, clinical remission was achieved by approximately one-third of patients at both 14 weeks and 12 months. An important treatment goal in the management of patients with IBD is the achievement and maintenance of sustained CS-free clinical remission [1, 2, 111]. Approximately, one-quarter of patients with UC or CD achieved CS-free clinical remission at 14 weeks and 42% of patients with UC and 31% of patients with CD at 12 months. As patients comprising the 12-month cohort likely represent the earliest vedolizumab users, they could represent a more severe, treatment-refractory cohort (most are likely to have failed anti-TNFα treatment). According to RCT experiences, greater effectiveness should be achieved in biologic-naive patients. In a real-world setting, this trend could induce higher efficacy rates with continued and earlier use of the drug. Also, in our study, up to approximately one-third of patients with CD achieved clinical remission after week 14, suggesting potential benefits of therapeutic monitoring beyond this time point. Despite including patients with more complex disease versus RCTs, real-world clinical and CS-free clinical remission rates in UC and CD reported here are consistent with, and in some cases exceed, vedolizumab efficacy reported in the GEMINI trials [5–7]. Moreover, the findings suggest a similar treatment effect in UC and CD, despite including patients with more complex disease versus RCTs.

Subgroup analyses in UC and CD biologic-naive patients receiving vedolizumab demonstrated substantially improved remission rates versus the overall patient population. These results further strengthen evidence that vedolizumab demonstrates greater effectiveness in anti-TNFα-naive patients. Post hoc analyses of GEMINI data indicated greater 12-month remission rates in anti-TNFα-naive patients versus anti-TNFα therapy failures (GEMINI 2 [CD] 49 versus 28%) [112] or versus anti-TNFα-experienced patients (GEMINI 1 [UC] 47 versus 36%) [113]. Several real-world studies have demonstrated better outcomes with vedolizumab in anti-TNFα-naive versus anti-TNFα-experienced patients [24, 36, 38, 46, 114–116]. The results from the current study are consistent with these findings.

In the current study in both UC and CD, Swedish cohorts had higher clinical remission rates, whereas cohorts in Germany and Spain had lower remission rates. The differences in remission rates based on geography need to be interpreted with caution, however, because of the small number of studies in this analysis. Several characteristics of IBD patients, including epidemiology, phenotype, and genotype, are known to vary with geography [117]. Geographic differences in study population baseline characteristics [e.g., disease severity at vedolizumab initiation, disease duration, prior anti-TNFα use (and number of prior therapies)], national treatment guidelines, and IBD management patterns may also account for variations in remission rates across geographic locations in our study. This is an area worthy of further investigation, but is beyond the current analysis.

Five publications included in the current review reported on hospitalization rates both pre-vedolizumab (6–12 months before initiation) and post-vedolizumab (6 months after initiation); 4 studies [63, 100, 101, 110] reported a reduction in post-treatment hospitalization rates, whereas 1 study [99] reported no change in hospitalization rates. Furthermore, a recent study in biologic-naive patients (published after the prespecified date range for this review) reported lower rates of IBD-related surgery and hospitalizations at 6 and 12 months after the first infusion of vedolizumab compared with infliximab [62]. Additional studies on the long-term effects of vedolizumab treatment on hospitalization rates are warranted.

Mucosal healing is an important IBD therapy goal associated with sustained clinical remission, CS-free clinical remission, and reduced hospitalization and surgery rates [118, 119]. Recent “treat-to-target” draft clinical guidelines state that only patients with mucosal healing (absence of macroscopic signs of active inflammation) and no/very mild signs or symptoms should be considered as remitted [120]. Among larger studies (sample size ≥ 100) in our systematic review, more than half of patients with UC or CD achieved mucosal healing at 12 months; results for UC were better than reported in GEMINI 1 [5]. Although data were limited to 12 studies, the observed rates of mucosal healing over 12 months were greater than the combined rates of clinical remission, supporting previous reports of a lack of clear correlation between clinical symptom measures and bowel damage assessed by endoscopy/colonoscopy or diagnostic imaging modalities [121, 122]. Interim results from the LOVE-CD trial demonstrated that, of 74 patients who underwent endoscopy, endoscopic remission (defined as Simple Endoscopic Score for CD ≤ 3) was observed in 30% of patients at week 26 [123]. Patients with endoscopic response were shown to have higher median vedolizumab concentrations compared with endoscopic nonresponders [123]. Results from the phase 3b, open-label, VERSIFY study (NCT02425111) will provide additional insights into rates of mucosal healing in CD patients receiving vedolizumab (manuscript in progress) [124].

Dose escalation is used to address secondary loss of response to biologics in the clinical management of IBD [125]. The studies included here (*n* = 8) [23, 33, 37, 41, 46, 57, 75, 108] reported that 4–60% of patients required dose escalation up to week 54, with lower rates reported in biologic-naive patients (*n* = 2; range 0–20%). However, the highest rates of dose escalation (47–60%) were observed in more complex, treatment-refractory UC and CD patients who were included as part of a compassionate-use program [23] and thus are unlikely to be representative of the general IBD population receiving biologics. In 2 studies, dose-escalation rates were lower with vedolizumab than with anti-TNFα agents [57, 75]. Of the few studies reporting dose-escalation outcomes (*N* = 4), at least one-third of patients were able to recapture response [33, 37, 46, 108].

A meta-analysis of 9 studies comprising 1565 adult patients with UC or CD was recently published by Engel and colleagues [126]. Investigation of rates of clinical remission, clinical response, CS-free clinical remission, and safety demonstrated that vedolizumab is efficacious in UC and CD and has a favorable safety profile. Our study results corroborate their findings. The overall AE rate reported by Engel and colleagues was 30.6% (6 studies, *n* = 306) compared with a rate of 0–67% (23 studies, *n* = 2358) in the current study. Nasopharyngitis and arthralgia were among the most common AEs reported in both meta-analyses.

A notable point of differentiation between the current study and the report by Engel and colleagues is the comprehensiveness of the current study with inclusion of not only full-text articles but also congress abstracts, thus allowing for additional effectiveness data to be assessed at relevant treatment time points. The thorough and inclusive approach adopted for the current review facilitated additional subgroup analyses by geographic location and in patients with no prior therapy with biologics. The current analysis also reports outcomes not assessed by Engel and colleagues, including hospitalization, surgical rates post-vedolizumab initiation, dose-escalation rates, and subsequent outcomes.

Vedolizumab is a gut-selective integrin antagonist with no identified systemic immunosuppressive activity [5–7, 127–130]. Real-world safety data reported here are consistent with those from the GEMINI trials, with no new or unexpected safety signals [128]. This tolerability profile may help to improve treatment persistence [115, 116], thereby potentially positively affecting long-term outcomes. Postoperative complication rates in the current analysis ranged from 13 to 65% [89, 102, 131–133]. A recent meta-analysis assessing the impact of preoperative vedolizumab treatment on the rate of postoperative complications in real-world patients with IBD demonstrated no increased risk of postoperative infectious or total overall postoperative complications compared with either preoperative anti-TNFα therapy or no biologic therapy [134].

In addition to the limitations of real-world studies, the limitations of this meta-analysis include potential publication bias. Egger’s weighted regression statistic was calculated for only 1 analysis (CD at week 14) and in this case the *P* value suggested that bias was unlikely. The remaining analyses did not include enough studies (≥ 10) to allow an assessment of publication bias [52]. However, the inclusion of studies published as abstracts as in the current analysis may help minimize the risk of publication bias. A moderate to high degree of between-study statistical heterogeneity was detected in some analyses. Major contributory factors to this heterogeneity may include the different disease activity measures and variable thresholds used to assess clinical response and remission, which may impact the extrapolation of these findings to clinical practice. Nevertheless, the current meta-analysis attempted to address bias by combining study data using a weighted average based on sample size. Moreover, the consistency of evidence levels (i.e., most studies were level 4) did not allow for sensitivity analysis to be conducted by study quality. Finally, real-world data may be less stringent than RCT data, which are obtained by rigorous data collection and quality control of data integrity. However, real-world data provide greater insight into the effectiveness of vedolizumab in heterogenous and more complex patient populations that are more representative of clinical practice.

## Conclusions

The results from this meta-analysis of real-world data confirm the effectiveness of vedolizumab in inducing long-term clinical response, clinical remission, CS-free clinical remission, and mucosal healing in patients with moderate-to-severely active UC or CD. The safety data presented here support the positive long-term benefit–risk profile of vedolizumab in the treatment of IBD.

## Electronic supplementary material

Below is the link to the electronic supplementary material.

Supplementary material 1 (DOCX 2434 kb)
