# Supplementary material for: Systematic review with meta-analysis: real-world effectiveness and safety of vedolizumab in patients with inflammatory bowel disease
Source: J Gastroenterol. 2018 Jun 4;53(9):1048–64. doi: 10.1007/s00535-018-1480-0 (PMC6132930; doi:10.1007/s00535-018-1480-0)
Supplement: Supplementary file 1 — Supplementary material 1 (DOCX 2434 kb) [file 535_2018_1480_MOESM1_ESM.docx]

Systematic Review With Meta-Analysis: Real-World Effectiveness and Safety of Vedolizumab in Patients With Inflammatory Bowel Disease

SUPPLEMENTARY DATA CONTENT

**Table S1** Summary of real-world clinical response and remission rates reported in studies evaluating vedolizumab therapy in patients with ulcerative colitis or Crohn’s disease

| **Study** | **Outcome measure** | **Assessment time point** | **Clinical response, n (%)** | **Clinical remission, n (%)** | **CS-free remission, n (%)** |
| --- | --- | --- | --- | --- | --- |
| **Ulcerative colitis** | | | | | |
| Amiot et al. 2017 [[1](#_ENREF_1)]  N = 121 | Response: PMS ≤3 with a >30% decrease from baseline and a ≥1-point decrease (rectal bleeding subscale) or a rectal bleeding absolute score of 0 or 1 from baseline  Remission: PMS <3, stool frequency and rectal bleeding subscore ≤1 | 6 weeks | 50 (41) | 39 (32) | 26 (22) |
|  |  | 14 weeks | 69 (57) | 47 (39) | 43 (36) |
|  |  | 6 months | 65 (54) | 51 (42) | 49 (41) |
|  |  | 12 months | 61 (50) | 51 (42) | 49 (41) |
| Baumgart et al. 2016 [[2](#_ENREF_2)]  N = 115 | Response: PMS reduction ≥3 with a >30% decrease from baseline  Remission: PMS ≤1 and a rectal bleeding subscore of 0 | 6 weeks | 49 (43) | 13 (11) | 10 (9) |
|  |  | 14 weeks | 66 (57) | 27 (23) | 22 (19) |
| Chaparro et al. 2016 [[3](#_ENREF_3)]  N = 42 | NR | 14 weeks | 32 (76)^a^ | 13 (31)^a^ | NR |
| Chaudrey et al. 2016 [[4](#_ENREF_4)]  N = 12 | NR | 6 weeks | 8 (67) | NR | NR |
| Christensen et al. 2015 [[5](#_ENREF_5)]  N = 20^b^ | Response: reduction ≥3 in SCCAI  Remission: SCCAI ≤2 | 14 weeks | 10 (50) | 8 (40) | NR |
| Dulai et al. 2017 [[6](#_ENREF_6)]  N = 180 | Response: >50% reduction in symptoms  Remission: complete resolution of all symptoms | 6 months | 95 (53) | 67 (37) | 40 (22) |
|  |  | 12 months | 131 (73) | 92 (51) | 74 (41) |
| Eriksson et al. 2017 [[7](#_ENREF_7)]  N = 39 | Mayo score (metrics NR) | 12 months | 23 (59) | 25 (64) | 23 (59) |
| Hoog et al. 2016 [[8](#_ENREF_8)]^c^  N = 16 | PMS, PGA (metrics NR) | 6 months | 3 (19)^d^ | 8 (50) | NR |
| Kopylov et al. 2017 [[9](#_ENREF_9)]  N = 74 | Remission:^e^ PMS <2 or SCCAI <4 | 14 weeks | 32 (43) | 20 (28) | 18 (24) |
| Lenti et al. 2017 [[10](#_ENREF_10)]  N = 36 | NR | 12 months | 10 (28) | 16 (44) | NR |
| Mankongpaisarnrung et al. 2016 [[11](#_ENREF_11)]  N = 7 | NR | 6 weeks | NR | 5 (71) | NR |
| Pauwels et al. 2017 [[12](#_ENREF_12)]  N = 6 | Endoscopic response: MAYO score reduction ≥1  Endoscopic remission: MAYO score ≤1 | 12 months | 3 (50) | 3 (6) | NR |
| Samaan et al. 2017 [[13](#_ENREF_13)]^f^ | Response: reduction in SCCAI ≥3  Remission: SCCAI <3 | 14 weeks (n = 18) | 10 (56)^a^ | 7 (39) | 6 (33) |
|  |  | 6 months (n = 10) | 6 (60) | 5 (50) | 5 (50)^g^ |
|  |  | 12 months (n = 12) | NR | 7 (58) | 7 (58) |
| Shelton et al. 2015 [[14](#_ENREF_14)]^c^ | Response: SCCAI ≥3 or physician assessment of clinical response  Remission: SCCAI ≤2 or physician assessment of clinical remission | 6 weeks  (n = 40) | 18 (45) | 6 (15) | NR |
|  |  | 14 weeks (n = 58) | 31 (53) | 17 (29) | 12 (23)  n = 52 |
| Shivashankar et al. 2017 [[15](#_ENREF_15)]  N = 31 | NR | 14 weeks | 18 (58) | NR | NR |
| Stallmach et al. 2016 [[16](#_ENREF_16)]  N = 60 | Response: reduction in PMS ≥3 with a ≥30% decrease from baseline  Remission: Week 54 PMS ≤1 with a rectal bleeding subscore of 0 in UC | 6 months | 28 (47) | 11 (18) | 9 (15) |
|  |  | 12 months | 23 (38) | 15 (25) | 13 (22) |
| Ungar et al. 2016 [[17](#_ENREF_17)]  N = 25 | Remission: SCCAI <3 | 6 weeks | NR | 6 (24) | NR |
| Vivio et al. 2016 [[18](#_ENREF_18)]  N = 15^b^ | Remission: PMS ≤2 and no subscore >1 | 14 weeks | NR | 8 (53)^a^ | NR |
| Wright et al. 2017 [[19](#_ENREF_19)]  N = 8 | Response: improvement of mucosal inflammation on posttreatment endoscopy | 6 months | 6 | NR | NR |
|  |  | 12 months | 5 | NR | NR |
| Zezos et al. 2017 [[20](#_ENREF_20)]  N = 57 | Remission: PMS ≤2 | 6 months | NR | 28 (49) | 25 (44) |
| **Crohn’s disease** | | | | | |
| Abramowitz et al. 2016 [[21](#_ENREF_21)]  N = 30 | Response: decrease in HBI ≥3  Remission: HBI ≤4 | 14 weeks | 18 (60) | 11 (37) | NR |
| Amiot et al. 2017 [[1](#_ENREF_1)]  N = 173 | Response: decrease in HBI ≤3  Remission: HBI ≤4 | 6 weeks | 98 (57) | 54 (31) | 33 (19) |
|  |  | 14 weeks | 110 (64) | 63 (36) | 53 (31) |
|  |  | 6 months | 94 (54) | 55 (32) | 48 (28) |
|  |  | 12 months | 82 (47) | 52 (30) | 47 (27) |
| Baumgart et al. 2016 [[2](#_ENREF_2)]  N = 97 | Response: decrease in HBI ≥3  Remission: HBI ≤4 | 6 weeks | 64 (66) | 16 (16) | 11 (11) |
|  |  | 14 weeks | 59 (61) | 23 (24) | 19 (20) |
| Blum et al. 2016 [[22](#_ENREF_22)]^c^ | Response: improvement in HBI  Remission: HBI <5 | 14 weeks (n = 22) | 8 (36) | 4 (18) | NR |
|  |  | 6 months (n = 16) | 8 (50) | 5 (31) | NR |
| Chaparro et al. 2016 [[3](#_ENREF_3)]  N = 53 | NR | 14 weeks | 39 (74)^a^ | 10 (19) | NR |
| Chaudrey et al. 2016 [[4](#_ENREF_4)]  N = 51 | NR | 6 weeks | 31 (61) | NR | NR |
| Christensen et al. 2015 [[5](#_ENREF_5)]  N = 26^b^ | Response: decrease in HBI ≥3  Remission: HBI ≤4 | 14 weeks | 15 (58) | 10 (38) | NR |
| De Vos et al. 2016 [[23](#_ENREF_23)]  N = 79 | Response: reduction in CDAI >100 or decrease in HBI ≥3  Remission: CDAI <150 or HBI ≤4 | 14 weeks | 55 (70) | 27 (34) | NR |
| Dulai et al. 2016 [[24](#_ENREF_24)]  N = 212 | Response: ≥50% reduction in CD-related symptom activity or severity based on PGA  Remission: complete resolution of all CD-related symptoms | 6 weeks | 86 (41) | 23 (11) | 10 (9)  n = 117 |
|  |  | 6 months | 68 (32) | 38 (18) | 21 (18)  n = 117 |
|  |  | 12 months | 123 (58) | 74 (35) | 40 (34)  n = 117 |
| Eriksson et al. 2017 [[7](#_ENREF_7)]  N = 68 | HBI score (metrics NR) | 12 months | 36 (53) | 41 (60) | 37 (54) |
| Gils et al. 2016 [[25](#_ENREF_25)]  N = 27^b^ | Response: >50% decrease in CRP from baseline  Remission: CRP <5 mg/L | 6 weeks | 14 (52) | 8 (30) | NR |
| Glover et al. 2015 [[26](#_ENREF_26)]  N = 39 | Remission: any reduction in HBI score from before vedolizumab initiation to after 14 weeks of therapy | 14 weeks | NR | 7 (18) | NR |
| Hoog et al. 2016 [[8](#_ENREF_8)]^c^  N = 27^b^ | HBI, PGA (metrics NR) | 6 months | 10 (37)^a,d^ | 7 (26) | NR |
| Kopylov et al. 2017 [[9](#_ENREF_9)]  N = 130 | Remission:^e^ HBI <5 | 14 weeks | 69 (53) | 45 (35) | 38 (29) |
| Lenti et al. 2017 [[10](#_ENREF_10)]  N = 48 | NR | 12 months | 8 (17) | 6 (13) | NR |
| Pauwels et al. 2017 [[12](#_ENREF_12)]  N = 17 | Endoscopic response: SES-CD reduction ≥50%;  Rutgeerts reduction of ≥1 (postop CD)  Endoscopic remission: SES-CD <4;  Rutgeerts score ≤1 (postop CD) | 12 months | 6/16 (38) | 1 (6) | NR |
| Samaan et al. 2017 [[13](#_ENREF_13)]^†f^ | Response: decrease in HBI ≥3  Remission: HBI <5 | 14 weeks (n = 19) | 12 (63) | 7 (37) | 5 (26) |
|  |  | 6 months (n = 10) | 5 (50) | 5 (50) | 4 (40)^g^ |
|  |  | 12 months  (n = 10) | NR | 4 (40) | 3 (30) |
| Shelton et al. 2015 [[14](#_ENREF_14)]^c^ | Response: decrease in HBI ≥3 or physician assessment of clinical response  Remission: HBI ≤4 or physician assessment of clinical response | 6 weeks  (n = 42) | 25 (60) | 15 (36) | NR |
|  |  | 14 weeks (n = 88) | 43 (49) | 21 (24) | 16 (19) |
| Shivashankar et al. 2017 [[15](#_ENREF_15)0]  N = 120 | Response: HBI score ≤3 points | 14 weeks | 52 (43) | NR | NR |
| Stallmach et al. 2016 [[16](#_ENREF_16)]  N = 67 | Response: decrease in HBI ≥3  Remission: Week 54 HBI ≤4 | 6 months | 17 (25) | 13 (19) | 9 (13) |
|  |  | 12 months | 17 (25) | 14 (21) | 10 (15) |
| Ungar et al. 2016 [[17](#_ENREF_17)]  N = 47 | Remission: HBI <5 | 6 weeks | NR | 14 (30) | NR |
| Wright *et al*. 2017 [[19](#_ENREF_19)]  N = 2 | Response: improvement of mucosal inflammation on posttreatment endoscopy | 6 months | 1 (50) | NR | NR |
|  |  | 12 months | 1 (50) | NR | NR |

*CD* Crohn’s disease, *CDAI* Crohn’s Disease Activity Index, *CRP* C-reactive protein, *CS* corticosteroid, *HBI* Harvey-Bradshaw Index, *NR* not reported, *PGA* physician global assessment, *PMS* partial Mayo Score, *SCCAI* Simple Clinical Colitis Activity Index, *SES-CD* simple endoscopic score in Crohn’s disease, *UC* ulcerative colitis

^a^ N not reported. Data calculated based on percentage of patients, with fraction rounded up

^b^ Numbers represent subset of patients with active disease analyzed in the study

^c^ Data derived from poster presentation

^d^ Partial response

^e^ PGA used when clinical scores were unavailable

^f^ Additional data derived from posters and author, including unpublished clinical data provided courtesy of Dr. Mark A. Samaan and Dr. Peter Irving from their UK study, 2016

^g^ Unpublished data provided by authors

**Table S2** Oxford Centre for Evidence-Based Medicine Levels of Evidence (2011) [[27](#_ENREF_27)]

| **Question** | **Step 1 (Level 1^a^)** | **Step 2  (Level 2^a^)** | **Step 3 (Level 3^a^)** | **Step 4 (Level 4^a^)** | **Step 5 (Level 5)** |
| --- | --- | --- | --- | --- | --- |
| How common is the problem? | Local and current random sample surveys (or censuses) | Systematic review of surveys that allow matching to local circumstances^b^ | Local non-random sample^b^ | Case series^b^ | N/A |
| Is this diagnostic or monitoring test accurate?  (Diagnosis) | Systematic review of cross-sectional studies with consistently applied reference standard and blinding | Individual cross-sectional studies with consistently applied reference standard and blinding | Non-consecutive studies or studies without consistently applied reference standards^b^ | Case-control studies, or “poor or non-independent” reference standard^b^ | Mechanism-based reasoning |
| What will happen if we do not add a therapy?  (Prognosis) | Systematic review of inception cohort studies | Inception cohort studies | Cohort study or control arm of randomised trial^a^ | Case series or case-control studies, or poor quality prognostic cohort study | N/A |
| Does this intervention help?  (Treatment benefits) | Systematic review of randomised trials or n‑of‑1 trials | Randomised trial or observational study with dramatic effect | Non-randomised controlled cohort/follow-up study^b^ | Case series, case-control studies, or historically controlled studies^b^ | Mechanism-based reasoning |
| What are the COMMON harms?  (Treatment harms) | Systematic review of randomised trials, systematic review of nested case-control studies, n‑of‑1 trial with the patient question being asked, or observational study with dramatic effect | Individual randomised trial or (exceptionally) observational study with dramatic effect | Non-randomised controlled cohort/follow-up study (post-marketing surveillance), provided there are sufficient numbers to rule out a common harm (for long-term harms, the duration of follow-up must be sufficient^b^) | Case series, case-control, or historically controlled studies^b^ | Mechanism-based reasoning |
| What are the RARE harms?  (Treatment harms) | Systematic review of randomised trials or n‑of‑1 trial | Randomised trial or (exceptionally) observational study with dramatic effect |  |  |  |
| Is this (early detection) test worthwhile?  (Screening) | Systematic review of randomized trials | Randomised trial | Non-randomised controlled cohort/follow-up study^b^ | Case series, case-control, or historically controlled studies^b^ | Mechanism-based reasoning |

*N/A* not available, *PICO* Patient, Problem or Population; Intervention; Comparison, Control or Comparator; Outcome

^a^ Level may be graded down on the basis of study quality, imprecision, or indirectness (study objectives do not match study question PICO), because of inconsistency between studies, or because the absolute effect size is very small; level may be graded up if there is a large or very large effect size

^b^ As always, a systematic review provides stronger evidence than an individual study

**Fig. S1** PRISMA diagram showing results of the systematic literature search

*CS* corticosteroid


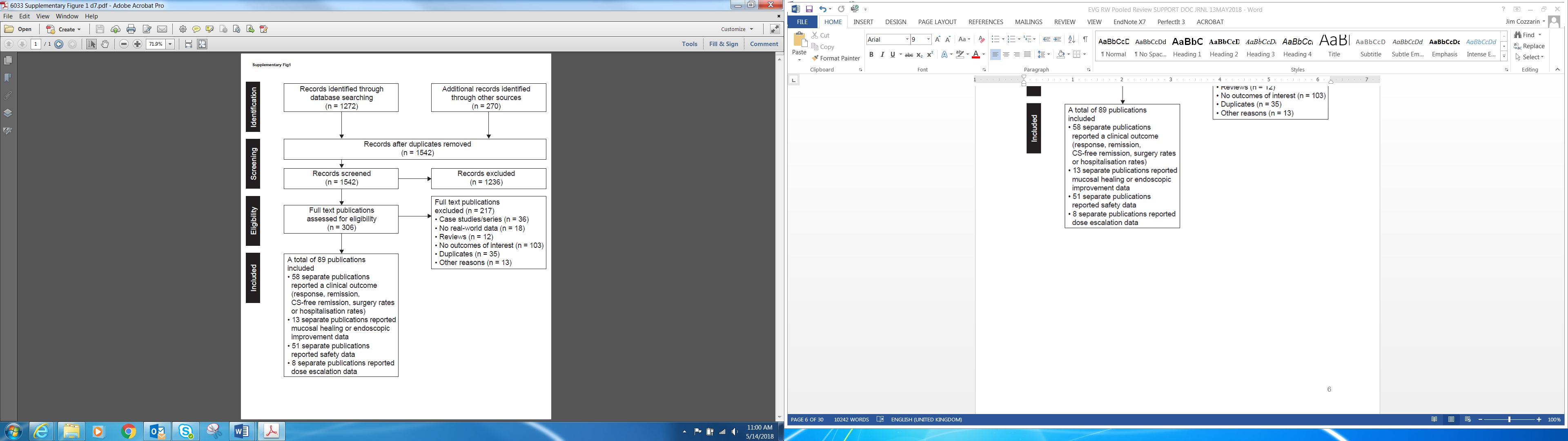


**Table S3** Study characteristics and patient demographics from studies included in the systematic literature review

| **Study** | **Level of evidence^a^** | **Country** | **Indication** | **Sample size, N** | **Mean age, years (±SD)** | **Male sex, %** | **Mean disease duration, years** | **Prior  5-ASA therapy, %^b^** | **Prior  IMS therapy, %^b^** | **Prior  CS  therapy, %^b^** | **Prior  TNF antagonist therapy, %^b^** | **Concomitant IMS  therapy, %^b^** | **Concomitant CS  therapy, %^b^** | **Concomitant IMS and CS therapy, %^b^** | **Fistulizing disease, %** | **Prior surgery, %** |
| --- | --- | --- | --- | --- | --- | --- | --- | --- | --- | --- | --- | --- | --- | --- | --- | --- |
| Abramowitz et al. 2016 [[21](#_ENREF_21)] | 4 | US | CD | 30 | NR | 47 | 18 | NR | NR | NR | 83 | NR | NR | NR | NR | NR |
| Aelvoet et al. 2016 [[28](#_ENREF_28)] | 4 | US | UC | 9 | NR | NR | NR | NR | NR | NR | NR | NR | NR | NR | NR | 100 |
|  |  |  | CD | 14 |  |  |  |  |  |  |  |  |  |  |  |  |
| Alam et al. 2016 [[29](#_ENREF_29)] | 4 | US | UC | 44 | 45 (±16.5) | 59.1 | NR | NR | NR | 59.1 | 0 | NR | 34.1 | NR | NR | 2 |
|  |  |  | CD | 78 | 47 (±16.7) | 48.7 |  |  |  | 60.3 | 0 |  | 33.3 |  |  | 14 |
| Alam et al. 2017 [[30](#_ENREF_30)] | 3 | US | UC | 30 | 44^c^ (NR) | 48.1 | 3.6^§^ | NR | NR | 60.5 | NR | NR | NR | NR | NR | 18.5 |
|  |  |  | CD | 51 |  |  |  |  |  |  |  |  |  |  |  |  |
| Allegretti et al. 2017 [[31](#_ENREF_31)] | 4 | US | UC | 40 | NR | NR | NR | NR | NR | NR | NR | NR | NR | NR | NR | NR |
|  |  |  | CD | 96 |  |  |  |  |  |  |  |  |  |  |  |  |
| Amiot et al. 2016 [[32](#_ENREF_32)] | 4 | France | UC | 121 | 42.8 (±16.1) | 55 | 9 | NR | 95 | NR | 98 | 12 | 34 | 10 | NR | NR |
|  |  |  | CD | 173 | 37.3 (±11.8) | 37 | 12 | NR | 97 | NR | 99 | 15 | 34 | 10 | NR | NR |
| Amiot et al. 2017 [[1](#_ENREF_1)] | 3 | France | UC | 111 | 41.8^c^ (NR) | 55 | 8.4^c^ | NR | 95.5 | NR | 98.2 | 18.0 | 15.3 | 2.7 | NR | NR |
|  |  |  | CD | 161 | 35.6^c^ (NR) | 37.3 | 10.7^c^ |  | 97.5 |  | 99.4 | 18.6 | 15.5 | 6.8 |  |  |
| Baumgart et al. 2016 [[2](#_ENREF_2)] | 4 | Germany | UC | 115 | 42^§^ | 57 | 7^§^ | NR | NR | NR | 76 | 77 | 84 | 64 | NR | 4 |
|  |  |  | CD | 97 | 36^§^ | 29 | 9^§^ | NR | NR | NR | 95 | 80 | 85 | 62 | NR | 42 |
| Bhayat et al. 2017 [[33](#_ENREF_33)] | 4 | UK | IBD | 406 | NR | NR | NR | NR | NR | NR | NR | NR | NR | NR | NR | NR |
| Blum et al. 2016 [[22](#_ENREF_22)] | 4 | NR | CD | 26 | 47 (±15.1) | 54 | NR | NR | NR | NR | NR | NR | NR | NR | NR | NR |
| Bownik et al. 2015 [[34](#_ENREF_34)] | 4 | US | UC | 10 | NR | 55 | NR | NR | NR | NR | 90 | 39 | 50 | NR | NR | 47 |
|  |  |  | CD | 26 |  |  |  |  |  |  |  |  |  |  | NR |  |
| Buer et al. 2017 [[35](#_ENREF_35)] | 4 | Norway | UC | 10 | NR | NR | NR | NR | NR | NR | NR | NR | NR | NR | NR | NR |
|  |  |  | CD | 5 |  |  |  |  |  |  |  |  |  |  |  |  |
| Chaparro et al. 2016 [[3](#_ENREF_3), [36](#_ENREF_36)] | 4 | Spain | UC | 42 | 44 (±14) | 43 | 11 | NR | 93 | NR | NR | 40 | NR | NR | NR | 33 |
|  |  |  | CD | 53 |  |  |  |  |  |  |  |  |  |  | 28 |  |
| Chaudrey et al. 2016 [[37](#_ENREF_37)] | 3 | US | IBD | 326 | 38 (±15) | NR | NR | NR | NR | NR | NR | NR | NR | NR | NR | NR |
| Chaudrey et al. 2016 [[4](#_ENREF_4)] | 4 | US | UC | 12 | NR | NR | NR | NR | NR | NR | 100 | 17 | 66 | NR | NR | NR |
|  |  |  | CD | 51 |  |  |  |  |  |  | 96 | 17 | 53 |  |  |  |
| Christensen et al. 2015 [[5](#_ENREF_5)] | 4 | US | UC | 27 | 21^§^ | 45 | NR | NR | NR | NR | 86 | NR | NR | NR | NR | NR |
|  |  |  | CD | 42 |  |  |  |  |  |  |  |  |  |  | NR |  |
| Christensen et al. 2015 [[38](#_ENREF_38)] | 4 | US | UC | 14 | 22^§^ | 46 | 9.5 | NR | NR | NR | NR | NR | NR | NR | NR | NR |
|  |  |  | CD | 23 |  |  |  |  |  |  |  |  |  |  |  |  |
| Christensen et al. 2017 [[39](#_ENREF_39)] | 4 | NR | IBD-PSC | 34 | NR | NR | NR | NR | NR | NR | NR | NR | NR | NR | NR | NR |
| Christopher et al. 2016 [[40](#_ENREF_40)] | 4 | UK | UC | 6 | 36 (NR) | 37 | NR | NR | NR | NR | 100 | 68 | NR | NR | NR | 42 |
|  |  |  | CD | 13 |  |  |  |  |  |  |  |  |  |  |  |  |

| **Study** | **Level of evidence^a^** | **Country** | **Indication** | **Sample size, N** | **Mean age, years (±SD)** | **Male sex, %** | **Mean disease duration, years** | **Prior  5-ASA therapy, %^b^** | **Prior  IMS therapy, %^b^** | **Prior  CS  therapy, %^b^** | **Prior  TNF antagonist therapy, %^b^** | **Concomitant IMS  therapy, %^b^** | **Concomitant CS  therapy, %^b^** | **Concomitant IMS and CS therapy, %^b^** | **Fistulizing disease, %** | **Prior surgery, %** |
| --- | --- | --- | --- | --- | --- | --- | --- | --- | --- | --- | --- | --- | --- | --- | --- | --- |
| Crowell et al. 2016 [[41](#_ENREF_41)] | 4 | US | CD | 42 | 39.3 (±10.9) | 38 | 10^c^ | NR | NR | >10 | >62 | NR | NR | NR | NR | NR |
| De Vos et al. 2016 [[23](#_ENREF_23)] | 4 | Belgium | UC | 42 | 40.7 (±13.4) | 59 | 13 | NR | NR | NR | 100 | NR | 54 | NR | NR | NR |
|  |  |  | CD | 79 |  | 38 |  |  |  |  |  |  |  |  |  |  |
| Drvarov et al. 2015 [[42](#_ENREF_42)] | 4 | NR | UC | 35 | 39 (NR) | NR | NR | NR | NR | NR | 100 | NR | NR | NR | NR | NR |
|  |  |  | CD | 43 | 38 (NR) |  |  |  | NR | NR | 100 | NR | NR |  | NR |  |
| Dulai *et al*. 2015 [[43](#_ENREF_43)]^d^, 2016 [[24](#_ENREF_24)], 2017 [[6](#_ENREF_6)] | 3 | US | CD | 212 | 34^c^ | 40 | 11^§^ | NR | NR | NR | 91 | 23 | 45 | 31 | 40 | 55 |
|  |  |  | UC | 180 | NR | 52 | NR | NR | NR | NR | NR | NR | 38 | NR | NR | NR |
| Eksteen et al. 2016 [[44](#_ENREF_44)] | 4 | NR | UC | 17 | NR | NR | NR | NR | NR | NR | NR | NR | NR | NR | NR | NR |
|  |  |  | CD | 8 |  |  |  |  |  |  |  |  |  |  | NR |  |
| Ehehalt et al. 2016 [[45](#_ENREF_45)] | 4 | Germany | UC | 32 | 41.6 | 50.9 | 10.3 | NR | NR | NR | NR | NR | NR | NR | NR | NR |
|  |  |  | CD | 21 |  |  |  |  |  |  |  |  |  |  |  |  |
| Eriksson et al. 2017 [[7](#_ENREF_7)] | 4 | Sweden | UC | 80 | 41.5^c^ (NR) | 59 | NR | NR | NR | NR | NR | NR | 38 | NR | NR | NR |
|  |  |  | CD | 120 | 42^c^ (NR) | 50 |  |  |  |  |  |  |  |  |  |  |
| Gabriëls et al. 2016 [[46](#_ENREF_46)] | 4 | The Netherlands | UC | 21 | 44 | 40 | 9.4 | NR | NR | NR | 90 | NR | NR | NR | NR | NR |
|  |  |  | CD | 27 |  |  |  |  |  |  |  |  |  |  |  |  |
| Gils et al. 2016 [[25](#_ENREF_25)] | 4 | Belgium | UC | 26 | NR | NR | NR | NR | NR | NR | NR | NR | NR | NR | NR | NR |
|  |  |  | CD | 49 |  |  |  |  |  |  |  |  |  |  | NR |  |
| Gils et al. 2017 [[47](#_ENREF_47)] | 4 | Belgium | UC | 29 | NR | NR | NR | NR | NR | NR | NR | NR | 38 | NR | NR | NR |
|  |  |  | CD | 46 |  |  |  |  |  |  |  |  |  |  |  |  |
| Glover et al. 2015 [[26](#_ENREF_26)] | 4 | US | CD | 39 | NR | NR | NR | 72 | 82 | 100 | 87 | NR | NR | NR | NR | NR |
| Grace et al. 2015 [[48](#_ENREF_48)] | 3 | US | UC/ID | 17 | 18-49 | NR | NR | NR | NR | NR | 92 | 36 | 50 | NR | NR | NR |
|  |  |  | CD | 41 |  |  |  |  |  |  |  |  |  |  |  |  |
|  |  |  | UC/ID | 8 | ≥50 |  |  |  |  |  | 80 | 50 | 50 |  |  |  |
|  |  |  | CD | 13 |  |  |  |  |  |  |  |  |  |  |  |  |
| Gudsoorkar et al. 2015 [[49](#_ENREF_49)] | 4 | US | CD | 20 | 38^§^ | 25 | 14^c^ | NR | NR | NR | NR | 60 | NR | NR | 60 | 65 |
| Hoog et al. 2016 [[8](#_ENREF_8)] | 4 | Sweden | UC | 16 | NR | NR | NR | NR | 100 | NR | 100 | NR | NR | NR | NR | NR |
|  |  |  | CD | 30 |  |  |  |  |  |  |  |  |  |  | NR |  |
| Kaimakliotis et al. 2017 [[50](#_ENREF_50)] | 4 | US | UC | 23 | 40 (±12.9) | 47.1 | 11.7 | NR | NR | NR | 91.4 | 42.8 | 54.2 | NR | NR | NR |
|  |  |  | CD | 35 |  |  |  |  |  |  |  |  |  |  |  |  |
|  |  |  | IBD-U | 10 |  |  |  |  |  |  |  |  |  |  |  |  |
| Kamperidis et al. 2017 [[51](#_ENREF_51)] | 4 | UK | UC | 22 | 39.8 (±14.1) | NR | NR | NR | NR | 36 | 55 | NR | NR | NR | NR | NR |
|  |  |  | CD | 15 | 36.6 (±13.3) |  |  |  |  | 33 | 100 |  |  |  |  | 60 |
| Kassim et al. 2017 [[52](#_ENREF_52)] | 4 | US | UC | 11 | 35.6 (NR) | 40.9 | NR | NR | NR | NR | NR | NR | NR | NR | NR | NR |
|  |  |  | CD | 11 |  |  |  |  |  |  |  |  |  |  |  |  |
| Khalid et al. 2016 [[53](#_ENREF_53)] | 4 | US | IBD | 101 | 43 | 39.6 | NR | NR | NR | NR | NR | NR | NR | NR | NR | NR |

| **Study** | **Level of evidence^a^** | **Country** | **Indication** | **Sample size, N** | **Mean age, years (±SD)** | **Male sex, %** | **Mean disease duration, years** | **Prior  5-ASA therapy, %^b^** | **Prior  IMS therapy, %^b^** | **Prior  CS  therapy, %^b^** | **Prior  TNF antagonist therapy, %^b^** | **Concomitant IMS  therapy, %^b^** | **Concomitant CS  therapy, %^b^** | **Concomitant IMS and CS therapy, %^b^** | **Fistulizing disease, %** | **Prior surgery, %** |
| --- | --- | --- | --- | --- | --- | --- | --- | --- | --- | --- | --- | --- | --- | --- | --- | --- |
| Kochhar et al. 2017 [[54](#_ENREF_54)] | 3 | US | UC | 214 | 40 (±17) | 51.4 | NR | NR | NR | NR | 68.7 | 30.8 | 54.7 | NR | NR | NR |
|  |  |  | CD | 293 | 38 (±15) | 41.3 |  |  |  |  | 91.8 | 38.6 | 52.9 |  |  |  |
| Koh et al. 2016 [[55](#_ENREF_55)] | 4 | US | UC | 6 | 29^§^ | 40 | NR | NR | NR | NR | NR | NR | NR | NR | NR | 100 |
|  |  |  | CD | 8 |  |  |  |  |  |  |  |  |  |  |  |  |
|  |  |  | IBD-U | 1 |  |  |  |  |  |  |  |  |  |  |  |  |
| Koliani-Pace et al.  2017 [[56](#_ENREF_56)] | 4 | US | UC | 306 | NR | NR | NR | NR | NR | NR | NR | NR | NR | NR | NR | NR |
|  |  |  | CD | 436 |  |  |  |  |  |  |  |  |  |  |  |  |
| Kopylov et al. 2016 [[57](#_ENREF_57)] | 4 | Israel | UC/IBD-U | 35/8 | 34.3 (NR) | NR | 8 | NR | NR | NR | 95 | NR | NR | NR | NR | NR |
|  |  |  | CD | 67 | 39.6 (±17.7) |  | 13 |  |  |  |  |  |  |  |  |  |
| Kopylov et al. 2017 [[9](#_ENREF_9)] | 4 | Israel | CD | 130 | 40.5 (±14.9) | 52.3 | 11 | NR | NR | NR | 96.9 | 24.6 | 40.8 | NR | NR | 35.4 |
|  |  |  | UC/IBD-U | 69/5 | 39.6 (±13.7) | 56.8 | 10 |  |  |  | 85.2 | 21.6 | 48.6 |  |  | NR |
| Kopylov et al. 2017 [[58](#_ENREF_58)] | 4 | Italy, Israel, Germany, France, UK, Finland | UC | 44 | 46 (±18.5) | 50.0 | NR | NR | NR | NR | NR | 25.0 | 38.6 | NR | NR | NR |
|  |  |  | CD | 13 | 45 (±18) | 69.2 | NR | NR | NR | NR | NR | 15.4 | 15.4 | NR | NR | NR |
| Lenti et al. 2017 [[10](#_ENREF_10)] | 4 | UK | CD | 120 | 41 (NR) | NR | NR | NR | NR | NR | 85 | 31 | 37 | NR | NR | NR |
|  |  |  | UC | 61 |  |  |  |  |  |  |  |  |  |  |  |  |
|  |  |  | IBD-U | 2 |  |  |  |  |  |  |  |  |  |  |  |  |
| Lenti et al. 2017 [[59](#_ENREF_59)] | 3 | UK | UC | 13 | 41 (NR) | NR | NR | NR | NR | NR | NR | NR | NR | NR | NR | NR |
| Lightner et al. 2017 [[60](#_ENREF_60)] | 4 | US | UC | 22 | 34^c^ | 29 | NR | NR | NR | NR | NR | 39 | 39 | NR | NR | 100 |
|  |  |  | CD | 71 |  |  |  |  |  |  |  |  |  |  |  |  |
|  |  |  | IBD-U | 1 |  |  |  |  |  |  |  |  |  |  |  |  |
| Lightner et al. 2017 [[61](#_ENREF_61)] | 4 | US | IBD | 142 | NR | NR | NR | NR | NR | NR | NR | NR | NR | NR | NR | NR |
| Lucci et al. 2015 [[62](#_ENREF_62)] | 4 | NR | UC | 12 | 38 (±3) | 37 | 31 | NR | NR | NR | NR | NR | NR | NR | NR | 57 |
|  |  |  | CD | 48 |  |  | 19 |  |  |  |  |  |  |  | 40 |  |
|  |  |  | IBD-U | 2 |  |  | NR |  |  |  |  |  |  |  | NR |  |
| Mahadevan et al.  2017 [[63](#_ENREF_63)] | 4 | US, EU, Canada | IBD | 81 | NR | NR | NR | NR | NR | NR | NR | NR | NR | NR | NA | NR |
| Mankong-paisarnrung et al. 2016 [[11](#_ENREF_11)] | 4 | US | UC | 7 | NR | NR | NR | NR | NR | NR | NR | NR | NR | NR | NA | NR |
|  |  |  | CD | 11 |  |  |  |  |  |  |  |  |  |  |  |  |
| Menon et al. 2016 [[64](#_ENREF_64)] | 4 | Australia | UC | 12 | 41 | 48 | 8 | NR | NR | NR | 81 | 43 | NR | NR | NR | NR |
|  |  |  | CD | 9 |  |  |  |  |  |  |  |  |  |  |  |  |
| Meserve et al. 2017 [[65](#_ENREF_65)] | 4 | NR | IBD | 259 | NR | NR | NR | NR | NR | NR | NR | NR | NR | NR | NR | NR |
| Morganstern et al. 2015 [[66](#_ENREF_66)] | 4 | US | UC | 17 | 30.5^c^ | NR | 8.6^§^ | NR | NR | NR | 92 | 37 | NR | NR | NR | 22 |
|  |  |  | CD | 45 |  |  |  |  |  |  |  |  |  |  |  |  |
|  |  |  | IBD-U | 4 |  |  |  |  |  |  |  |  |  |  |  |  |
| Navaneethan et al. 2016 [[67](#_ENREF_67), [68](#_ENREF_68)] | 4 | US | UC | 10 | 67.1 (±5.3) | NR | NR | NR | NR | NR | 69 | NR | NR | NR | NR | NR |
|  |  |  | CD | 19 |  |  |  |  |  |  |  |  |  |  |  |  |

| **Study** | **Level of evidence^a^** | **Country** | **Indication** | **Sample size, N** | **Mean age, years (±SD)** | **Male sex, %** | **Mean disease duration, years** | **Prior  5-ASA therapy, %^b^** | **Prior  IMS therapy, %^b^** | **Prior  CS  therapy, %^b^** | **Prior  TNF antagonist therapy, %^b^** | **Concomitant IMS  therapy, %^b^** | **Concomitant CS  therapy, %^b^** | **Concomitant IMS and CS therapy, %^b^** | **Fistulizing disease, %** | **Prior surgery, %** |
| --- | --- | --- | --- | --- | --- | --- | --- | --- | --- | --- | --- | --- | --- | --- | --- | --- |
| Oppenheim et al. 2015 [[69](#_ENREF_69)] | 4 | US | UC | 10 | 42 (NR) | 40 | 15.3 | NR | 90 | NR | 93 | 30 | 50 | NR | NR | NR |
|  |  |  | CD | 20 |  |  |  |  |  |  |  |  |  |  | NR |  |
| Papamichail et al. 2015 [[70](#_ENREF_70)] | 3 | Belgium, Greece, France | UC | 123 | NR | NR | NR | NR | NR | NR | 100 | NR | NR | NR | NR | NR |
|  |  |  | CD | 75 |  |  |  |  |  |  |  |  |  |  |  |  |
| Patel et al. 2016 [[71](#_ENREF_71)] | 4 | US | UC | 15 | NR | NR | NR | NR | NR | NR | NR | NR | NR | NR | NR | NR |
|  |  |  | CD | 25 |  |  |  |  |  |  |  |  |  |  | NR |  |
| Pauwels et al. 2017 [[12](#_ENREF_12)] | 4 | The Netherlands | UC | 58 | 39 (NR) | 41 | 11^§^ | NR | NR | NR | 98 | 31 | 76 | NR | NR | NR |
| Peerani et al. 2016 [[72](#_ENREF_72)] | 3 | US | UC | 114 | 39.5 (±15.5) | 60 | 8.9 | NR | NR | NR | 71 | 36 | 65 | NR | NR | NR |
| Plevris et al. 2017 [[73](#_ENREF_73)] | 4 | Scotland | CD | 27 | NR | NR | 7.9^c^ | NR | NR | NR | 57 | NR | NR | NR | NR | NR |
|  |  |  | UC | 33 |  |  |  |  |  |  |  |  |  |  |  |  |
|  |  |  | IBD-U | 3 |  |  |  |  |  |  |  |  |  |  |  |  |
| Raluy-Callado et al. 2016 [[74](#_ENREF_74)] | 4 | US | UC | 39 | 45 (±16.2) | 48.7 | 2.9 | 38.5 | 33.3 | 87.2 | 0 | NR | 33.3 | NR | 0 | NR |
|  |  |  | CD | 73 |  | 41.1 | 4.3 | 26.0 | 42.5 | 74 |  |  | 27.4 |  | 21.9 |  |
| Raluy Callado et al. 2016 [[75](#_ENREF_75)] | 4 | US | UC | 33 | 44 | 46.2 | 4.1^c^ | 34.1 | 17.6 | 59.3 | NR | NR | NR | NR | 4.4 | 8 |
|  |  |  | CD | 58 |  |  |  |  |  |  |  |  |  |  |  |  |
| Reynolds et al. 2016 [[76](#_ENREF_76)] | 4 | US | UC | 69 | 43 (±15.7) | 38 | 5.2 | NR | NR | 44.3 | NR | NR | NR | NR | 0 | 38 |
|  |  |  | CD | 168 |  |  |  |  |  |  |  |  |  |  | 7.1 |  |
| Reynolds et al. 2016 [[77](#_ENREF_77)] | 4 | US | UC | 101 | 43 (±15.3) | 42.6 | 3.1 | 8.9 | 8.9 | 51.5 | NR | NR | NR | NR | NR | NR |
|  |  |  | CD | 252 | 44 (±14.7) | 36.5 | 5.4 | 4 | 19 | 49.2 |  |  |  |  | 11.5 |  |
| Robinson et al. 2017 [[78](#_ENREF_78)] | 4 | UK | IBD | 55 | NR | NR | NR | NR | NR | NR | NR | NR | NR | NR | NR | NR |
| Samaan et al. 2017 [[13](#_ENREF_13)]^e^ | 4 | UK | UC | 20 | 37^§^ | 48 | 6^§^ | NR | NR | NR | 76 | 42 | NR | NR | NR | NR |
|  |  |  | CD | 27 |  |  |  |  |  |  |  |  |  |  |  |  |
|  |  |  | IBD-U | 3 |  |  |  |  |  |  |  |  |  |  |  |  |
| Schils et al. 2017 [[79](#_ENREF_79)] | 4 | Belgium | CD | 12 | 31 | 25 | 12 | NR | NR | NR | NR | NR | NR | NR | NR | NR |
| Schmidt et al. 2017 [[80](#_ENREF_80)] | 4 | Germany | UC | 60 | NR | NR | NR | NR | NR | NR | 81.7 | NR | 53.4 | NR | NR | NR |
| Shelton et al. 2015 [[14](#_ENREF_14)] | 4 | US | UC | 59 | 40.5 (±13.7) | 49 | 10 | NR | NR | NR | >62 | 26 | 58 | NR | NR | NR |
|  |  |  | IBD-U | 6 |  |  |  |  |  |  |  |  |  |  |  |  |
|  |  |  | CD | 107 | 39.7 (±14) | 48 | 16 | NR | NR | NR | >77 | 32 | 39 | NR | 15^f^ | 59 |
| Shivashankar et al. 2017 [[15](#_ENREF_15), [81](#_ENREF_81)] | 4 | US | UC | 31 | 40 (NR) | 43.1 | NR | NR | NR | NR | NR | 33 | 51 | NR | NR | NR |
|  |  |  | CD | 120 |  |  |  |  |  |  |  |  |  |  |  |  |
| Stallmach et al. 2016 [[16](#_ENREF_16)] | 4 | Germany | UC | 60 | 40.5^c^ | 58 | 10^§^ | NR | NR | NR | 68 | 22 | 47 | 7 | NR | 5 |
|  |  |  | CD | 67 | 37^c^ | 46 | 7^§^ | NR | NR | NR | 87 | 15 | 57 | 6 | Perianal: 22  Other: 8 | 52 |
| Stevens et al. 2015 [[82](#_ENREF_82)] | 4 | US | UC | 20 | 39 (NR) | 60 | 12 | NR | NR | NR | 100 | 33 | 67 | NR | NR | NR |
|  |  |  | CD | 22 |  |  |  |  |  |  |  |  |  |  | NR |  |
| Stringfield et al. 2016 [[83](#_ENREF_83)] | 4 | US | IBD | 26 | NR | NR | NR | NR | NR | NR | NR | NR | NR | NR | NR | 100 |

| **Study** | **Level of evidence^a^** | **Country** | **Indication** | **Sample size, N** | **Mean age, years (±SD)** | **Male sex, %** | **Mean disease duration, years** | **Prior  5-ASA therapy, %^b^** | **Prior  IMS therapy, %^b^** | **Prior  CS  therapy, %^b^** | **Prior  TNF antagonist therapy, %^b^** | **Concomitant IMS  therapy, %^b^** | **Concomitant CS  therapy, %^b^** | **Concomitant IMS and CS therapy, %^b^** | **Fistulizing disease, %** | **Prior surgery, %** |
| --- | --- | --- | --- | --- | --- | --- | --- | --- | --- | --- | --- | --- | --- | --- | --- | --- |
| Tadbiri et al. 2017 [[84](#_ENREF_84)] | 4 | France | UC | 121 | NR | NR | NR | NR | NR | NR | NR | NR | NR | NR | 17.3 | NR |
|  |  |  | CD | 173 |  |  |  |  |  |  |  |  |  |  |  |  |
| Trefois et al. 2016 [[85](#_ENREF_85)] | 4 | Belgium | UC | 9 | 47.5^c^ (±22.3^g^) | 30 | 11.3^§^ | NR | NR | NR | 80 | NR | 35 | NR | 25 | 40 |
|  |  |  | CD | 10 |  |  |  |  |  |  |  |  |  |  |  |  |
|  |  |  | IBD-U | 1 |  |  |  |  |  |  |  |  |  |  |  |  |
| Turkeltaub et al. 2017 [[86](#_ENREF_86)] | 4 | NR | Special IBD population^h^ | 47 | 47.7 (NR) | NR | 13.6 | NR | NR | NR | 51.1 | NR | NR | NR | NR | NR |
|  |  |  | IBD | 204 | 37.3 (NR) |  | 10.8 |  |  |  | 97.1 |  |  |  |  |  |
| Ungar et al. 2016 [[17](#_ENREF_17)] | 3 | Israel | UC | 25 | NR | NR | NR | NR | NR | NR | NR | NR | NR | NR | NR | NR |
|  |  |  | CD | 47 |  |  |  |  |  |  |  |  |  |  |  |  |
| Vivio et al. 2016 [[18](#_ENREF_18)] | 4 | US | UC | 21 | 46.2 (NR) | 38 | NR | NR | 29 | NR | 76 | 48 | 48 | NR | NR | 5 |
|  |  |  | CD | 30 | 49 (NR) | 47 |  | NR | 53 | NR | 97 | 70 | 67 | NR | NR | 47 |
| Wice et al. 2016 [[87](#_ENREF_87)] | 4 | US | UC | 25 | 43 (NR) | 43 | 9 | NR | 87 | NR | 96 | 34 | NR | NR | NR | NR |
|  |  |  | CD | 52 | 43 (NR) |  | 14 |  |  |  |  |  |  |  | NR | 53 |
| Williamson et al. 2017 [[88](#_ENREF_88)] | 4 | UK | IBD-PSC | 11 | 27 | 91% | NR | NR | NR | NR | NR | NR | NR | NR | NR | NR |
| Wright et al. 2017 [[19](#_ENREF_19)] | 4 | US | UC | 8 | 44^c^ (NR) | 80 | NR | NR | NR | NR | 40 | NR | 100 | NR | NR | NR |
|  |  |  | CD | 2 |  |  |  |  |  |  |  |  |  |  |  |  |
| Ylisaukko-oja et al. 2017 [[89](#_ENREF_89)] | 4 | Finland | UC | 127 | 38.2 (14.2) | 57 | NR | NR | NR | NR | 95 | NR | 62 | NR | NR | NR |
|  |  |  | CD | 105 | 39.8 (13.6) | 44 |  |  |  |  | 97 |  | 44 |  |  |  |
| Zezos et al. 2017 [[20](#_ENREF_20)] | 4 | Canada | UC | 57 | 33^§^ (NR) | 68 | 6^§^ | NR | 53 | NR | 68 | 18 | 46 | 11 | NR | NR |

*5-ASA* 5-aminosalicylic acid, *CD* Crohn’s disease, *CS* corticosteroid, *EU* European Union, *IBD* inflammatory bowel disease, *IBD-PSC* inflammatory bowel disease associated with primary sclerosing cholangitis, *IBD-U* IBD unspecified, *ID* indeterminate disease, *IMS* immunosuppressant, *NR* not reported, *SD* standard deviation, *TNF* tumor necrosis factor, *UC* ulcerative colitis, *UK* United Kingdom, *US* United States

^a^ Based on the Oxford Centre for Evidence-Based Medicine 2011 Levels of Evidence criteria [[27](#_ENREF_27)]

^b^ The highest percentage of patients taking any one of the medications is reported

^c^ Median, not mean

^d^ Data for both UC and CD are described in this publication

^e^ Unpublished clinical data provided courtesy of Dr. Mark A. Samaan and Dr. Peter Irving from their UK study, 2016

^f^ Data only available for 46/107 CD patients

^g^ Interquartile range

^h^ Includes 12 organ transplant recipients, 5 with neurologic disease, 4 with cirrhosis, 19 with increased susceptibility to infection, and 7 with active or recent malignancy

**Fig. S2** Summary of real-world endoscopic improvement rates among patients with ulcerative colitis (a) or Crohn’s disease (b) receiving vedolizumab

*CD* Crohn’s disease, *CI* confidence interval, *UC* ulcerative colitis

Squares represent individual studies, with the size of the square representing the sample size. Error bars represent 95% CIs.

Data from Christensen et al. [[38](#_ENREF_38)], Vivio et al. [[18](#_ENREF_18)], Pauwels et al. [[12](#_ENREF_12)], Williamson et al. [[88](#_ENREF_88)]

^a^ Median time point

^b^ Mean follow-up time

^c^ Only patients with ≥1 follow-up assessment at the specified time point were included in the analyses.
Data from the VICTORY Consortium, which contributed the majority of mucosal healing data, used a cumulative incidence analysis, while remaining studies employed a ‘complete’ case approach

For UC, mucosal healing (described as endoscopic remission in one study) [[38](#_ENREF_38)] was defined as a Mayo endoscopic score of 0–1, and endoscopic improvement as a ≥1-point reduction in the Mayo endoscopic score from baseline (patients achieving mucosal healing were also regarded as showing endoscopic improvement in one study) [[18](#_ENREF_18)]. For CD, mucosal healing was defined as absence of mucosal ulcers and/or erosions (excluding one study, which defined mucosal healing as a CD Endoscopic Index of Severity [CDEIS] score <3) [[38](#_ENREF_38)] and endoscopic improvement was defined in one study as a >50% mean change in CDEIS score [[38](#_ENREF_38)] and in another study as the endoscopist’s final impression of visible CD activity compared with baseline [[18](#_ENREF_18)].


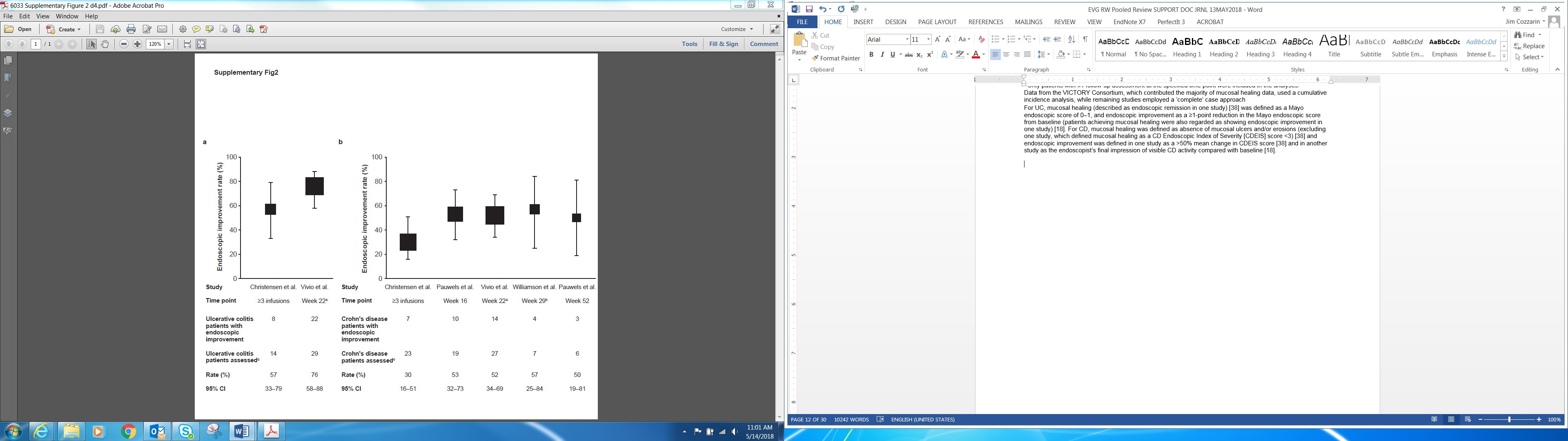


**Table S4** Safety of vedolizumab therapy in real-world studies: (a) overall and (b) individual adverse event rates

**a**

| **Event** | **No. of studies** | **Total patients, n** | **Range, %** | | **References** |
| --- | --- | --- | --- | --- | --- |
|  |  |  | **Min** | **Max** |  |
| Adverse events | 23 | 2,358 | 0 | 67 | Allegretti et al. [[31](#_ENREF_31)], Amiot et al. [[32](#_ENREF_32)], Bownik et al. [[34](#_ENREF_34)], Chaparro et al. [[36](#_ENREF_36)], Christensen et al. [[5](#_ENREF_5)], Christopher et al. [[40](#_ENREF_40)], Crowell et al. [[41](#_ENREF_41)], Drvarov et al. [[42](#_ENREF_42)], Gudsoorkar et al. [[49](#_ENREF_49)], Kaimakliotis et al. [[50](#_ENREF_50)], Kopylov et al. [[9](#_ENREF_9)], Lenti et al. [[10](#_ENREF_10)], Mahadevan et al. [[63](#_ENREF_63)], Meserve et al. [[65](#_ENREF_65)], Navaneethan et al. [[67](#_ENREF_67)], Patel et al. [[71](#_ENREF_71)], Shelton et al. [[14](#_ENREF_14)], Shivashankar et al. [[15](#_ENREF_15), [81](#_ENREF_81)], Stallmach et al. [[16](#_ENREF_16)], Stevens et al. [[82](#_ENREF_82)], Trefois et al. [[85](#_ENREF_85)], Ylisaukko-oja et al. [[89](#_ENREF_89)], Zezos et al. [[20](#_ENREF_20)] |
| Serious adverse events^a^ | 4 | 857 | 0 | 13 | Amiot et al. [[32](#_ENREF_32)], Chaudrey et al. [[37](#_ENREF_37)], Dulai et al. [[24](#_ENREF_24)], Eksteen et al. [[44](#_ENREF_44)] |
| Postoperative adverse events^b^ | 4 | 147 | 8 | 65 | Koh et al. [[55](#_ENREF_55)], Lightner et al. [[60](#_ENREF_60)], Stringfield et al. [[83](#_ENREF_83)], Schils et al. [[79](#_ENREF_79)] |
| Postoperative serious adverse events^c^ | 1 | 14 | 43 | | Aelvoet et al. [[28](#_ENREF_28)] |
| Infections | 12 | 1,176 | 5 | 24 | Amiot et al. [[32](#_ENREF_32)], Bownik et al. [[34](#_ENREF_34)], Chaudrey et al. [[4](#_ENREF_4)], Dulai et al. [[43](#_ENREF_43)], Eksteen et al. [[44](#_ENREF_44)], Grace et al. [[48](#_ENREF_48)], Kaimakliotis et al. [[50](#_ENREF_50)], Shelton et al. [[14](#_ENREF_14)], Shivashankar et al. [[81](#_ENREF_81)], Stallmach et al. [[16](#_ENREF_16)], Turkeltaub et al. [[86](#_ENREF_86)], Wright et al. [[19](#_ENREF_19)] |
| Serious infections^a^ | 3 | 832 | 4 | 10 | Amiot et al. [[32](#_ENREF_32)], Chaudrey et al. [[37](#_ENREF_37)], Dulai et al. [[24](#_ENREF_24)] |

^a^ Serious adverse events/infections were defined as any adverse event/infection resulting in disability or persistent damage, colectomy [[32](#_ENREF_32)], discontinuation/interruption of vedolizumab, hospitalization, death [[24](#_ENREF_24), [32](#_ENREF_32), [37](#_ENREF_37)] or any infection requiring antibiotics [[24](#_ENREF_24), [37](#_ENREF_37)]

^b^ Within 30 days postoperative

^c^ Within 4 weeks postoperative

**b**

| **Adverse event** | **No. of studies** | **Total patients, n** | **Range, %** | | **References** |
| --- | --- | --- | --- | --- | --- |
|  |  |  | **Min** | **Max** |  |
| **Non-infectious events** | | | | | |
| Arthralgia | 12 | 1,540 | <1 | 20 | Amiot et al. [[32](#_ENREF_32)], Blum et al. [[22](#_ENREF_22)], Chaparro et al. [[36](#_ENREF_36)], Dulai et al. [[24](#_ENREF_24)], Kaimakliotis et al. [[50](#_ENREF_50)], Kopylov et al. [[9](#_ENREF_9)], Patel et al. [[71](#_ENREF_71)], Plevris et al. [[73](#_ENREF_73)], Robinson et al. [[78](#_ENREF_78)], Shelton et al. [[14](#_ENREF_14)], Shivashankar et al. [[81](#_ENREF_81)], Stallmach et al. [[16](#_ENREF_16)] |
| Headache | 10 | 1,029 | <1 | 10 | Amiot et al. [[32](#_ENREF_32)], Bownik et al. [[34](#_ENREF_34)], Gudsoorkar et al. [[49](#_ENREF_49)], Kopylov et al. [[9](#_ENREF_9)], Menon et al. [[64](#_ENREF_64)], Patel et al. [[71](#_ENREF_71)], Shivashankar et al. [[81](#_ENREF_81)], Stallmach et al. [[16](#_ENREF_16)], Wice et al. [[87](#_ENREF_87)], Zezos et al. [[20](#_ENREF_20)] |
| Fatigue | 5 | 357 | 1 | 19 | Chaparro et al. [[36](#_ENREF_36)], Eksteen et al. [[44](#_ENREF_44)], Kaimakliotis et al. [[50](#_ENREF_50)], Patel et al. [[71](#_ENREF_71)], Stallmach et al. [[16](#_ENREF_16)] |
| Rash | 7 | 1,078 | <1 | 4 | Baumgart et al. [[2](#_ENREF_2)], Chaudrey et al. [[37](#_ENREF_37)], Lenti et al. [[10](#_ENREF_10)], Shelton et al. [[14](#_ENREF_14)], Vivio et al. [[18](#_ENREF_18)], Wice et al. [[87](#_ENREF_87)], Zezos et al. [[20](#_ENREF_20)] |
| Exacerbation of IBD symptoms | 4 | 645 | <1 | 14 | Amiot et al. [[32](#_ENREF_32)], Baumgart et al. [[2](#_ENREF_2)], Christensen et al. [[5](#_ENREF_5)], Kaimakliotis et al. [[50](#_ENREF_50)] |
| Fever | 5 | 574 | <1 | 3 | Baumgart et al. [[2](#_ENREF_2)], Kopylov et al. [[9](#_ENREF_9)], Oppenheim et al. [[69](#_ENREF_69)], Vivio et al. [[18](#_ENREF_18)], Wice et al. [[87](#_ENREF_87)] |
| Other gastrointestinal tract-related^a^ | 4 | 666 | <1 | 10 | Baumgart et al. [[2](#_ENREF_2)], Dulai et al. [[24](#_ENREF_24)], De Vos et al. [[23](#_ENREF_23)], Kaimakliotis et al. [[50](#_ENREF_50)], Shelton et al. [[14](#_ENREF_14)] |
| Infusion reaction | 9 | 1,023 | 0 | 5 | Amiot et al. [[32](#_ENREF_32)], Bownik et al. [[34](#_ENREF_34)], Chaparro et al. [[36](#_ENREF_36)], Dulai et al. [[24](#_ENREF_24)], Kopylov et al. [[9](#_ENREF_9)], Navaneethan et al. [[68](#_ENREF_68)], Robinson et al. [[78](#_ENREF_78)], Shelton et al. [[14](#_ENREF_14)], Shivashankar et al. [[81](#_ENREF_81)] |
| Nausea | 4 | 411 | <1 | 10 | Kaimakliotis et al. [[50](#_ENREF_50)], Shelton et al. [[14](#_ENREF_14)], Stallmach et al. [[16](#_ENREF_16)], Stevens et al. [[82](#_ENREF_82)] |
| Other skin and subcutaneous-related^b^ | 6 | 907 | <1 | 11 | Amiot et al. [[32](#_ENREF_32)], Buer et al. [[35](#_ENREF_35)], Chaparro et al. [[36](#_ENREF_36)], Kopylov et al. [[9](#_ENREF_9)], Shelton et al. [[14](#_ENREF_14)], Stallmach et al. [[16](#_ENREF_16)] |
| Allergic reactions | 3 | 190 | 1 | 2 | Christensen et al. [[5](#_ENREF_5)], Kaimakliotis et al. [[50](#_ENREF_50)], Vivio et al. [[18](#_ENREF_18)] |
| Other liver-related^c^ | 6 | 932 | <1 | 15 | Amiot et al. [[32](#_ENREF_32)], Christensen et al. [[39](#_ENREF_39)], Kopylov et al. [[9](#_ENREF_9)], Lenti et al. [[10](#_ENREF_10)], Morganstern et al. [[66](#_ENREF_66)], Shivashankar et al. [[81](#_ENREF_81)] |
| Other nervous system-related^d^ | 4 | 844 | <1 | 5 | Amiot et al. [[32](#_ENREF_32)], Chaudrey et al. [[37](#_ENREF_37)], Gudsoorkar et al. [[49](#_ENREF_49)], Kopylov et al. [[9](#_ENREF_9)] |
| Paresthesia | 3 | 526 | <1 | 5 | Amiot et al. [[32](#_ENREF_32)], Baumgart et al. [[2](#_ENREF_2)], Gudsoorkar et al. [[49](#_ENREF_49)] |
| Pruritus | 5 | 754 | <1 | 3 | Amiot et al. [[32](#_ENREF_32)], Baumgart et al. [[2](#_ENREF_2)], Patel et al. [[71](#_ENREF_71)], Shivashankar et al. [[15](#_ENREF_15)], Zezos et al. [[20](#_ENREF_20)] |
| Acne/acne-like lesions | 2 | 290 | 7 | 8 | Baumgart et al. [[2](#_ENREF_2)], Drvarov et al. [[42](#_ENREF_42)] |
| Autoimmune hepatitis | 2 | 538 | <1 | | Chaudrey et al. [[37](#_ENREF_37)], Dulai et al. [[24](#_ENREF_24)] |
| Cough | 2 | 185 | <1 | 13 | Baumgart et al. [[2](#_ENREF_2)], Kaimakliotis et al. [[50](#_ENREF_50)] |
| Dizziness | 2 | 222 | 2 | 7 | Chaparro et al. [[36](#_ENREF_36)], Stallmach et al. [[16](#_ENREF_16)] |
| Ear and labyrinth-related^e^ | 2 | 498 | <1 | 2 | Amiot et al. [[32](#_ENREF_32)], Kopylov et al. [[9](#_ENREF_9)] |
| Hives | 2 | 189 | 1 | 3 | Bownik et al. [[34](#_ENREF_34)], Shivashankar et al. [[81](#_ENREF_81)] |
| Memory impairment | 2 | 233 | <1 | 5 | Baumgart et al. [[2](#_ENREF_2)], Menon et al. [[64](#_ENREF_64)] |
| Cancer | 3 | 439 | <1 | 2 | Amiot et al. [[32](#_ENREF_32)], Plevris et al. [[73](#_ENREF_73)], Vivio et al. [[18](#_ENREF_18)] |
| Muscle pains | 1 | 70 | 4 | | Kaimakliotis et al. [[50](#_ENREF_50)] |
| Other musculoskeletal and connective tissue-related^f^ | 5 | 884 | <1 | 11 | Buer et al. [[35](#_ENREF_35)], Chaudrey et al. [[37](#_ENREF_37)], Shelton et al. [[14](#_ENREF_14)], Tadbiri et al. [[84](#_ENREF_84)], Wice et al. [[87](#_ENREF_87)] |
| Optic neuritis | 2 | 538 | <1 | | Chaudrey et al. [[37](#_ENREF_37)], Dulai et al. [[24](#_ENREF_24)] |
| Malnutrition | 1 | 212 | <1 | | Baumgart et al. [[2](#_ENREF_2)] |
| Night sweats | 1 | 70 | 14 | | Kaimakliotis et al. [[50](#_ENREF_50)] |
| Acute renal failure | 1 | 212 | <1 | | Oppenheim et al. [[69](#_ENREF_69)] |
| Spontaneous nose bleed | 1 | 70 | 14 | | Kaimakliotis et al. [[50](#_ENREF_50)] |
| Deep venous thrombosis | 1 | 294 | <1 | | Amiot et al. [[32](#_ENREF_32)] |
| **Infectious events** | | | | | |
| Other upper respiratory tract infection^g^ | 12 | 1193 | 1 | 21 | Amiot et al. [[32](#_ENREF_32)], Bownik et al. [[34](#_ENREF_34)], Buer et al. [[35](#_ENREF_35)], Gudsoorkar et al. [[49](#_ENREF_49)], Kaimakliotis et al. [[50](#_ENREF_50)], Kopylov et al. [[9](#_ENREF_9)], Lenti et al. [[10](#_ENREF_10)], Menon et al. [[64](#_ENREF_64)], Oppenheim et al. [[69](#_ENREF_69)], Patel et al. [[71](#_ENREF_71)], Shivashankar et al. [[81](#_ENREF_81)], Stallmach et al. [[16](#_ENREF_16)] |
| *Clostridium difficile* | 8 | 1,065 | <1 | 20^h^ | Amiot et al. [[32](#_ENREF_32)], Bownik et al. [[34](#_ENREF_34)], Dulai et al. [[24](#_ENREF_24)], Kopylov et al. [[9](#_ENREF_9)], Navaneethan et al. [[68](#_ENREF_68)], Shivashankar et al. [[81](#_ENREF_81)], Stallmach et al. [[16](#_ENREF_16)], Wright et al. [[19](#_ENREF_19)] |
| Other gastrointestinal infections^i^ | 9 | 1,438 | <1 | 6 | Amiot et al. [[32](#_ENREF_32)], Baumgart et al. [[2](#_ENREF_2)], Christensen et al. [[39](#_ENREF_39)], Dulai et al. [[24](#_ENREF_24)], Kaimakliotis et al. [[50](#_ENREF_50)], Kopylov et al. [[9](#_ENREF_9)], Lenti et al. [[10](#_ENREF_10)], Shelton et al. [[14](#_ENREF_14)], Zezos et al. [[20](#_ENREF_20)] |
| Other respiratory infections^j^ | 3 | 299 | 2 | 4 | Chaparro et al. [[36](#_ENREF_36)], Stallmach et al. [[16](#_ENREF_16)], Wice et al. [[87](#_ENREF_87)] |
| Genitourinary tract infections^k^ | 5 | 658 | <1 | 7 | Dulai et al. [[24](#_ENREF_24)], Kaimakliotis et al. [[50](#_ENREF_50)], Lenti et al. [[10](#_ENREF_10)], Morganstern et al. [[66](#_ENREF_66)], Stallmach et al. [[16](#_ENREF_16)] |
| Herpes zoster | 2 | 307 | 1 | 1 | Baumgart et al. [[2](#_ENREF_2)], Chaparro et al. [[36](#_ENREF_36)] |
| Sinusitis | 3 | 576 | 1 | 17 | Amiot et al. [[32](#_ENREF_32)], Dulai et al. [[24](#_ENREF_24)], Kaimakliotis et al. [[50](#_ENREF_50)] |
| Flu or flu-like infections | 3 | 450 | 1 | 7 | Amiot et al. [[32](#_ENREF_32)], Navaneethan et al. [[68](#_ENREF_68)], Stallmach et al. [[16](#_ENREF_16)] |
| Other infections^l^ | 4 | 342 | 1 | 3 | Bownik et al. [[34](#_ENREF_34)], Kaimakliotis et al. [[50](#_ENREF_50)], Lenti et al. [[10](#_ENREF_10)], Vivio et al. [[18](#_ENREF_18)] |
| Perianal abscess | 3 | 284 | 1 | 4 | Kaimakliotis et al. [[50](#_ENREF_50)], Shelton et al. [[14](#_ENREF_14)], Stevens et al. [[82](#_ENREF_82)] |
| Folliculitis | 3 | 246 | 1 | 3 | Bownik et al. [[34](#_ENREF_34)], Shivashankar et al. [[81](#_ENREF_81)], Zezos et al. [[20](#_ENREF_20)] |
| Meningitis | 3 | 546 | <1 | | Dulai et al. [[24](#_ENREF_24)], Lenti et al. [[10](#_ENREF_10)], Shivashankar et al. [[81](#_ENREF_81)] |
| Conjunctivitis | 1 | 51 | 2 | | Vivio et al. [[18](#_ENREF_18)] |
| **Postoperative events^m^** | | | | | |
| Postoperative septic shock and death | 1 | 212 | <1 | | Dulai et al. [[24](#_ENREF_24)] |
| Surgical site infections | 1 | 94 | 37 | | Lightner et al. [[60](#_ENREF_60)] |
| Non-surgical site infections | 1 | 94 | 7 | | Lightner et al. [[60](#_ENREF_60)] |
| Wound stitch abscess | 1 | 15 | 7 | | Koh et al. [[55](#_ENREF_55)] |
| Readmission for ileus | 1 | 15 | 7 | | Koh et al. [[55](#_ENREF_55)] |

*CMV* cytomegalovirus, *IBD* inflammatory bowel disease

^a^ Includes abdominal pain, anal fissure, aphthous ulcer, acute subobstruction, perforation during colonoscopy, bowel perforation, toothache, and complex fistulising disease

^b^ Includes paradoxical skin manifestations, acute generalized exanthematous pustulosis, dry skin, erythema nodosum, palmar erythema, and peristomal pyoderma gangrenosum

^c^ Includes liver test abnormalities, drug-induced liver injury (not specified), and transient transaminitis

^d^ Includes stroke, transverse myelitis (inflamed spinal cord), and lightheadedness

^e^ Includes vertigo and transient hearing loss

^f^ Includes severe musculoskeletal syndrome and exacerbation of pre-existing enteropathic arthritis

^g^ Includes upper respiratory tract infection (not specified), nasopharyngitis, and tonsillitis

^h^ Bownik et al. [[34](#_ENREF_34)] reported that in a small sample of very severe, treatment-refractory IBD patients receiving vedolizumab (n = 38), two patients (5%) developed a *Clostridium difficile* infection

^i^ Includes gastrointestinal infection (not specified), *Helicobacter* gastritis, CMV duodenitis, CMV colitis, *Campylobacter* infection, *Salmonella* gastroenteritis, abdominal abscess, and intra-abdominal abscess

^j^ Includes pneumonia, lower respiratory tract infections, and respiratory tract infection (not specified)

^k^ Includes urinary tract infection and urogenital infection

^l^ Includes histoplasmosis, methicillin-resistant *Staphylococcus aureus*, and bacteremia

^m^ Within 30 days postoperative

**Table S5** Meta-analysis of real-world clinical response and corticosteroid-free response rates among patients with ulcerative colitis or Crohn’s disease receiving vedolizumab

|  | **n** | **Clinical response,**  **% (95% CI)** | **N** | **CS-free response,**  **% (95% CI)** |
| --- | --- | --- | --- | --- |
| **Ulcerative colitis** | | | | |
| Week 6 | 288 | 43 (38–49) | 121 | 26 (19–35) |
| Week 14 | 479 | 56 (50–62) | 121 | 50 (40–59) |
| 6 months | 395 | 51 (43–59) | 301 | 40 (18–67) |
| 12 months | 450 | 52 (37–65) | 301 | 49 (43–54) |
| **Crohn’s disease** | | | | |
| Week 6 | 602 | 56 (46–65) | 290 | 28 (18–42) |
| Week 14 | 837 | 58 (51–64) | 173 | 51 (43–59) |
| 6 months | 507 | 40 (29–52) | 290 | 34 (19–54) |
| 12 months | 587 | 40 (29–52) | 290 | 46 (36–56) |
| *CI* confidence interval, *CS* corticosteroid  Data represent those derived from random-effects analyses | | | | |

**Table S6** Real-world IBD-related surgery and hospitalization rates reported in studies evaluating vedolizumab therapy in patients with ulcerative colitis or Crohn’s disease

| **Study** | **Indication** | **Time period** | **Sample size, n** | **IBD-related surgery rate, %** | **IBD-related hospitalization rate, %** |
| --- | --- | --- | --- | --- | --- |
| Alam et al. 2017 [[29](#_ENREF_29)]^a^ | UC | 6 months post vedolizumab initiation | 44 | 0 | 0 |
|  | CD | 6 months post vedolizumab initiation | 78 | 1 | 9 |
| Alam et al. 2017 [[30](#_ENREF_30)] | IBD | 12 months post vedolizumab initiation | 81 | NR | 12 |
| Allegretti et al. 2017 [[31](#_ENREF_31)] | IBD | Between 14–54 weeks post vedolizumab induction | 136 | NR | 40 |
| Amiot et al. 2017 [[1](#_ENREF_1)] | IBD | At 14 weeks post vedolizumab initiation | 272 | 3 | NR |
|  | IBD | Between 14–54 weeks post vedolizumab initiation | 272 | 3 | NR |
| Bhayat et al. 2017 [[33](#_ENREF_33)] | IBD (URTIs) | ≤30 days before event onset reported | 300 | 0 | NR |
|  | IBD (LRTIs) | ≤30 days before event onset reported | 106 | 2 | NR |
| Blum et al. 2016 [[22](#_ENREF_22)] | CD | 6 months post vedolizumab initiation | 26 | 4 | 8 |
| Buer et al. 2017 [[35](#_ENREF_35)] | UC | Mean follow-up: 9 (range, 5–2) months | 15 | 7 | NR |
| Chaudrey et al. 2016 [[4](#_ENREF_4)] | UC | NR (post induction) | 12 | 25 | NR |
|  | CD |  | 51 | 16 |  |
| Christensen et al. 2015 [[38](#_ENREF_38)] | IBD | NR | 39 | 18 | NR |
| Crowell et al. 2016 [[41](#_ENREF_41)] | CD | Median follow-up: 10 months post vedolizumab initiation | 42 | 24 | NR |
| De Vos et al. 2016 [[23](#_ENREF_23)] | IBD | Follow-up: 14 weeks post vedolizumab initiation | 121 | 7 | NR |
| Dulai et al. 2016 [[24](#_ENREF_24)] | CD | 6 months post vedolizumab initiation | 212 | 10 | NR |
|  |  | 12 months post vedolizumab initiation | 212 | 23 |  |
| Eksteen et al. 2016 [[44](#_ENREF_44)] | IBD | NR | 25 | 8 | NR |
| Gabriëls et al. 2016 [[46](#_ENREF_46)] | IBD | NR | 48 | 2 | NR |
| Kaimakliotis et al. 2016 [[50](#_ENREF_50)] | IBD | Median follow-up: 30 weeks post vedolizumab initiation | 70 | NR | 6 |
| Kamperidis et al. 2017 [[51](#_ENREF_51)] | UC | Mean follow-up: 8.2 (3.5) months | 22 | 5 | NR |
| Kassim et al. 2017 [[52](#_ENREF_52)] | IBD | Follow-up: ≥6 months | 57 | 4 |  |
| Koliani-Pace et al. 2017 [[56](#_ENREF_56)] | UC | Median follow-up: 10 months | 306 | 13 | NR |
|  | CD |  | 436 | 17 | NR |
| Kopylov et al. 2016 [[57](#_ENREF_57)] | UC | NR | 39 | 13 | 18 |
|  | CD |  | 67 | 13 | 22 |
| Kopylov et al. 2017 [[9](#_ENREF_9)] | IBD | 14 weeks post vedolizumab initiation | 204 | NR | 1 |
|  | IBD | 12 weeks post vedolizumab therapy+surgery | 130 | 6 | NR |
| Kopylov et al. 2017 [[58](#_ENREF_58)] | IBD | 14 weeks post vedolizumab initiation | 57 | NR | 5 |
| Lenti et al. 2017 [[59](#_ENREF_59)] | UC | 9 months | 13 | 31 | NR |
| Lightner et al. 2017 [[61](#_ENREF_61)] | IBD | 12 weeks post vedolizumab therapy+surgery | 142 | 7 | 18 |
| Lucci et al. 2015 [[62](#_ENREF_62)] | IBD | Median follow-up: 102 days post vedolizumab initiation | 62 | NR | 10 |
| Mahadevan et al. 2017 [[63](#_ENREF_63)] | IBD | 18 weeks post vedolizumab initiation | 24 | NR | 4 |
| Menon et al. 2016 [[64](#_ENREF_64)] | UC | NR | 12 | 0 | 0 |
|  | CD |  | 9 | 0 | 0 |
| Meserve et al. 2017 [[65](#_ENREF_65)] | IBD | Within 90 days of last vedolizumab infusion | 16 | 0 | 18 |
| Navaneethan et al. 2017 [[68](#_ENREF_68)] | IBD | After 12 months vedolizumab maintenance therapy | 29 | 10 | NR |
| Papamichail et al. 2015 [[70](#_ENREF_70)] | IBD | NR | 19 | 37 | NR |
| Patel et al. 2016 [[71](#_ENREF_71)] | UC | Follow-up: 16 months post vedolizumab initiation | 15 | 20 | NR |
|  | CD |  | 25 | 8 |  |
| Peerani et al. 2016 [[72](#_ENREF_72)] | UC | 6 months post vedolizumab initiation | 114 | 6 | NR |
|  |  | 12 months post vedolizumab initiation |  | 21 |  |
| Plevris et al. 2017 [[73](#_ENREF_73)] | IBD | 30 weeks post vedolizumab initiation | 94 | 7 | NR |
| Raluy-Callado et al. 2016 [[74](#_ENREF_74)]^a^ | UC | 6 months post vedolizumab initiation | 39 | 5 | 5 |
|  | CD |  | 73 | 6 | 6 |
| Raluy-Callado et al. 2016 [[75](#_ENREF_75)]^a^ | IBD | 6 months post vedolizumab initiation | 91 | NR | 8 |
| Reynolds et al. 2016 [[76](#_ENREF_76)]^a^ | IBD | 6 months post vedolizumab initiation | 237 | NR | 11 |
| Reynolds et al. 2016 [[77](#_ENREF_77)]^a^ | IBD | 6 months post vedolizumab initiation | 353 | NR | 15 |
| Samaan et al. 2017 [[13](#_ENREF_13)] | UC | 30 weeks post vedolizumab initiation | 23 | 4 | NR |
|  | CD |  | 27 | 11 | NR |
| Shelton et al. 2015 [[14](#_ENREF_14)] | IBD | 14 weeks post vedolizumab initiation | 193 | NR | 2 |
| Schils et al. 2017 [[79](#_ENREF_79)] | CD | <14 weeks post vedolizumab initiation | 12 | 100 | NR |
| Vivio et al. 2016 [[18](#_ENREF_18)] | UC | 52 weeks post vedolizumab initiation | 51 | 12 | NR |
|  | CD |  | 64 | 13 | NR |
| Wright et al. 2017 [[19](#_ENREF_19)] | IBD | 12 months post vedolizumab initiation | 10 | NR | 10 |
|  | UC | 6 months post vedolizumab initiation | 10 | 10 | NR |
|  | UC | 12 months post vedolizumab initiation | 10 | 10 | NR |
| Zezos et al. 2017 [[20](#_ENREF_20)] | UC | 2 months post vedolizumab initiation | 57 | 5 | NR |
|  |  | 4 months post vedolizumab initiation | 57 | 9 | NR |
|  |  | 6 months post vedolizumab initiation | 57 | 14 | NR |

*CD* Crohn’s disease, *IBD* inflammatory bowel disease, *LRTI* lower respiratory tract infection, *NR* not reported, *UC* ulcerative colitis, *URTI* upper respiratory tract infection

^a^ Studies from the EXPLORYS Universe cohort; data capture different time points, disease or treatment populations, or population sizes

**Table S7** Vedolizumab dose-escalation rates in patients with ulcerative colitis or Crohn’s disease

|  | **Indication** | **Time period (post vedolizumab initiation)** | **Sample size, n** | **Dose escalation,**  **% of patients** | **Outcomes post-dose escalation** |
| --- | --- | --- | --- | --- | --- |
| Amiot et al. 2017 [[1](#_ENREF_1)] | UC | 54 weeks | 121 | 47.0 | NR |
|  | CD |  | 173 | 60.0 |  |
| Dulai et al. 2016 [[24](#_ENREF_24)] | CD | 39 weeks (median) | 212 | 10.0 | Clinical response in 4 (30.8%) and clinical remission in 1 (7.7%) of  13 patients with lack of response or  suboptimal response  Clinical response regained in  3 (37.5%) and clinical remission in  1 (12.5%) of 8 patients with loss of response or remission |
| Ehehalt et al. 2016 [[45](#_ENREF_45)] | UC^†^ | Mean follow-up (SD): 1.4 (0.4) years | 12 | 17.0 | NR |
|  | CD^†^ |  | 5 | 20.0 |  |
|  | UC^‡^ |  | 21 | 29.0 |  |
|  | CD^‡^ |  | 16 | 13.0 |  |
| Khalid et al. 2016 [[53](#_ENREF_53)] | IBD (UC and CD)^a^ | 210 days | 28 | 4.0 | NR |
|  | IBD (UC and CD)^b^ |  | 68 | 6.0 |  |
| Mankongpaisarnrung et al. 2016 [[11](#_ENREF_11)] | CD | NR | 11 | 36.0 | 75% with minimal to mild clinical improvement |
| Shivashankar et al. 2017 [[15](#_ENREF_15)] | UC | 29.5 weeks (mean) | 31 | 16.0 | Of the patients who lost response,  81.4% (22/27) recaptured response by end of study |
|  | CD |  | 120 | 18.0 |  |
| Wice et al. 2016 [[87](#_ENREF_87)] | UC | 41 weeks (range, 4–19 months) (CD);  NR (UC) | 25 | 16.0 | Of the patients with CD, 64% (9/14) responded |
|  | CD |  | 52 | 27.0 |  |
| Wright et al. 2017 [[19](#_ENREF_19)] | IBD (UC and CD)^b^ | NR | 10 | 20.0 | NR |

*CD* Crohn’s disease, *IBD* inflammatory bowel disease, *NR* not reported, *SD* standard deviation, *UC* ulcerative colitis

Higher rates of dose escalation were observed in the treatment-refractory patients in the Amiot study [[32](#_ENREF_32)], all of whom were included as part of a compassionate use program.

^a^ Biologic-naive patients

^b^ Biologic-experienced patients

**Fig. S3** Meta-analysis clinical remission rates at Week 14 (a) and Month 12 (b) among biologic-naive patients with ulcerative colitis receiving vedolizumab

*CI* confidence interval

Square size represents the weight given to each study, based on sample size. Error bars represent 95% CIs. Diamonds represent the point estimate of the averaged study rates; lateral tips of the diamonds represent 95% CIs.

Data from Baumgart et al. [[2](#_ENREF_2)], Kopylov et al. [[9](#_ENREF_9)], Samaan et al. [[13](#_ENREF_13)], Dulai et al. [[6](#_ENREF_6)], Stallmach et al. [[16](#_ENREF_16)]


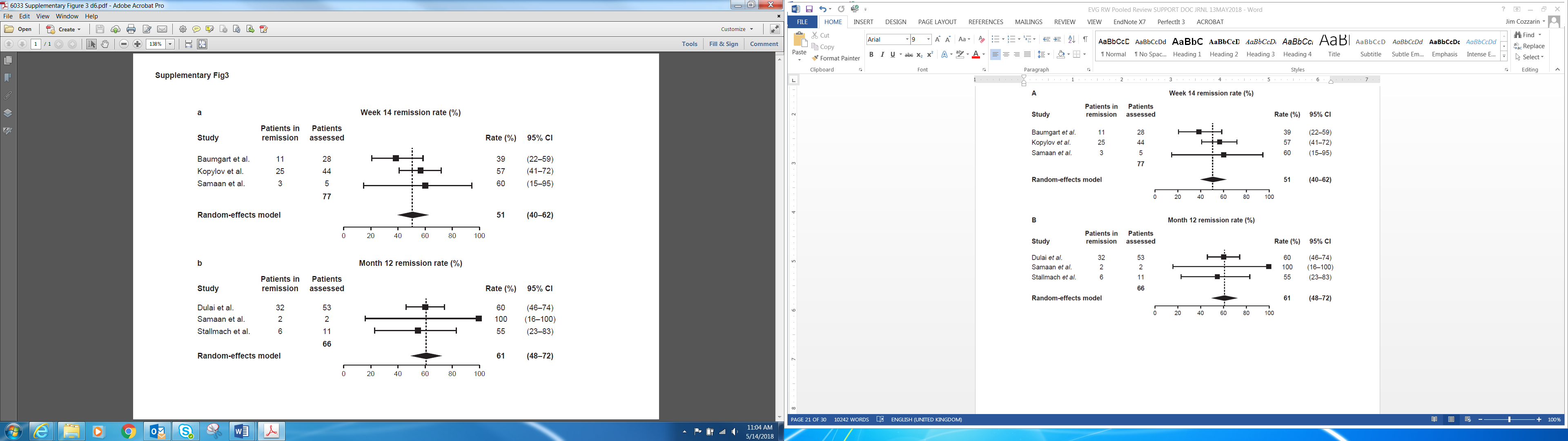


**Fig. S4** Meta-analysis of clinical remission rates at Week 14 (a) and Month 12 (b) among biologic-naive patients with Crohn’s disease receiving vedolizumab

*CI* confidence interval

Square size represents the weight given to each study, based on sample size. Error bars represent 95% CIs. Diamonds represent the point estimate of the averaged study rates; lateral tips of the diamonds represent 95% CIs.

Data from Baumgart et al. [[2](#_ENREF_2)], Kopylov et al. [[9](#_ENREF_9)], Samaan et al. [[13](#_ENREF_13)], Stallmach et al. [[16](#_ENREF_16)]


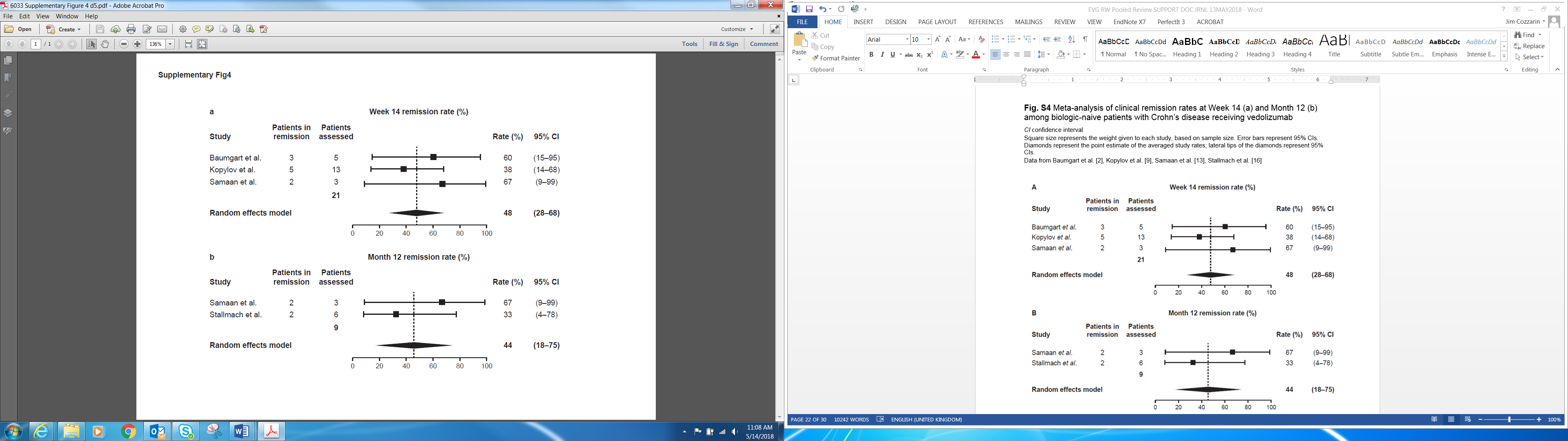


References

1. Amiot A, Serrero M, Peyrin-Biroulet L, et al. One-year effectiveness and safety of vedolizumab therapy for inflammatory bowel disease: a prospective multicentre cohort study. Aliment Pharmacol Ther*.* 2017;46(3):310–21.

2. Baumgart DC, Bokemeyer B, Drabik A, et al. Vedolizumab induction therapy for inflammatory bowel disease in clinical practice--a nationwide consecutive German cohort study. Aliment Pharmacol Ther*.* 2016;43(10):1090–102.

3. Chaparro M, Sierra-Ausin M, Mesonero F, et al. Effectiveness and safety of vedolizumab for the induction of remission in inflammatory bowel disease. J Crohns Colitis*.* 2016;10(suppl 1):S416–7.

4. Chaudrey K, Lightner A, Singh S, et al. Efficacy and safety of vedolizumab for inflammatory bowel disease in clinical practice. Inflamm Bowel Dis*.* 2016;22(suppl 1):S19–20.

5. Christensen B, Goeppinger SR, Colman RJ, et al. Vedolizumab in the treatment of IBD: the University of Chicago experience. Gastroenterology*.* 2015;148(4 suppl 1):S866.

6. Dulai P, Meserve J, Hartke J, et al. DOP023. Predictors of clinical and endoscopic response with vedolizumab for the treatment of moderately-severely active ulcerative colitis: results from the US VICTORY consortium. J Crohns Colitis*.* 2017;11(suppl 1):S40–1.

7. Eriksson C, Rundquist S, Lykiardopoulos B, et al. P364. A Swedish observational study (SVEAH) on vedolizumab assessing effectiveness and healthcare resource utilization in patients with inflammatory bowel disease. J Crohns Colitis*.* 2017;11(suppl 1):S262–3.

8. Höög C, Eberhardson M, Almer S. P419. Efficacy of vedolizumab in patients with inflammatory bowel disease and failure of anti-TNF-antibodies. Presented at: European Crohn’s and Colitis Organisation Congress. March 16–19, 2016; Amsterdam, The Netherlands.

9. Kopylov U, Ron Y, Avni-Biron I, et al. Efficacy and safety of vedolizumab for induction of remission in inflammatory bowel disease-the Israeli real-world experience. Inflamm Bowel Dis*.* 2017;23(3):404–8.

10. Lenti MV, Levison S, Eliadou E, et al. P525. Effectiveness and safety of vedolizumab in IBD patients: a multicentre experience of “real world data” from the UK. J Crohns Colitis*.* 2017;11(suppl 1):S347.

11. Mankongpaisarnrung C, Mattar M, Charabaty A. Single-center experience: vedolizumab in patients with Crohn's disease and ulcerative colitis at Georgetown University Hospital. Inflamm Bowel Dis*.* 2016;22(suppl 1):S32.

12. Pauwels RWM, De Vries AC, Van der Woude CJ. P447. Vedolizumab induces significantly higher endoscopic remission rates at week 16 in ulcerative colitis as compared to Crohn's disease. J Crohns Colitis*.* 2017;11(suppl 1):S305.

13. Samaan MA, Pavlidis P, Johnston E, et al. Vedolizumab: early experience and medium-term outcomes from two UK tertiary IBD centres. Frontline Gastroenterol. 2017;8(3):196–202.

14. Shelton E, Allegretti JR, Stevens B, et al. Efficacy of vedolizumab as induction therapy in refractory IBD patients: a multicenter cohort. Inflamm Bowel Dis*.* 2015;21(12):2879–85.

15. Shivashankar R, Mendoza Ladd AH, Grace R, et al. Effect of vedolizumab dose escalation on recapturing response in patients with inflammatory bowel disease. Gastroenterology*.* 2017;152(5 suppl 1):S77.

16. Stallmach A, Langbein C, Atreya R, et al. Vedolizumab provides clinical benefit over 1 year in patients with active inflammatory bowel disease – a prospective multicenter observational study. Aliment Pharmacol Ther*.* 2016;44(11–12):1199–212.

17. Ungar B, Kopylov U, Waterman M, et al. Early vedolizumab drug levels and induction success in patients with inflammatory bowel disease. United European Gastroenterol J*.* 2016;4(5 suppl):A3.

18. Vivio EE, Kanuri N, Gilbertsen JJ, et al. Vedolizumab effectiveness and safety over the first year of use in an IBD clinical practice. J Crohns Colitis*.* 2016;10(4):402–9.

19. Wright AP, Fontana RJ, Stidham RW. Vedolizumab is safe and effective in moderate-to-severe inflammatory bowel disease following liver transplantation. Liver Transpl. 2017;23(7):968–71.

20. Zezos P, Kabakchiev B, Weizman AV, et al. P502. Ulcerative colitis patients on vedolizumab lacking response at induction phase continue to improve over the first 6 months of treatment. J Crohns Colitis*.* 2017;11(suppl 1):S334–5.

21. Abramowitz M, Dale M, Saumoy M, et al. Harvey-Bradshaw index captures clinical efficacy of vedolizumab induction therapy for active Crohn's disease. Inflamm Bowel Dis*.* 2016;22(suppl 1):S27.

22. Blum A, Gregory N, Keyur P, et al. Vedolizumab for the treatment of Crohn’s disease: experience in an inflammatory bowel disease clinical practice. Am J Gastroenterol. 2016;111(suppl 1):S276.

23. De Vos M, Dhooghe B, Vermeire S, et al. P0868. Open label observational study evaluating the effect of induction therapy with vedolizumab in patients with moderate to severe inflammatory bowel disease intolerant/resistant to at least two biologicals. United European Gastroenterol J*.* 2016;4(5 suppl):A452.

24. Dulai PS, Singh S, Jiang X, et al. The real-world effectiveness and safety of vedolizumab for moderate-severe Crohn's disease: results from the US VICTORY Consortium. Am J Gastroenterol*.* 2016;111(8):1147–55.

25. Gils A, Dreesen E, Compernolle G, et al. Vedolizumab exposure correlates with clinical, biological and endoscopic outcome in patients with inflammatory bowel disease. United European Gastroenterol J*.* 2016;4(5 suppl):A3.

26. Glover S, Edminster T, Garcia L, et al. Efficacy of vedolizumab in Crohn's disease after at least 14 weeks of therapy as measured by HBI scores. Am J Gastroenterol. 2015;110(suppl 1):S826.

27. OCEBM Levels of Evidence Working Group. The Oxford 2011 Levels of Evidence. https://[www.cebm.net/wp-content/uploads/2014/06/CEBM-Levels-of-Evidence-2.1.pdf](http://www.cebm.net/wp-content/uploads/2014/06/CEBM-Levels-of-Evidence-2.1.pdf). Accessed March 15, 2017.

28. Aelvoet AS, Tran AL, Rubin DT. The influence of vedolizumab on postoperative outcomes in IBD patients undergoing abdominal surgery. Am J Gastroenterol. 2016;111:S297.

29. Alam N, Raluy-Callado M, Donaldson R, et al. P-066. Hospitalisations, flares, and corticosteroid use outcomes in biologic-naive patients with ulcerative colitis and Crohn's disease initiating vedolizumab. Inflamm Bowel Dis*.* 2017;23(suppl 1):S26–7.

30. Alam N, Raluy-Callado M, Gardstein B, et al. P470. Comparative effectiveness analysis of flares, hospitalisations, and corticosteroid use among biologic naïve patients with inflammatory bowel disease within 12 months of initiation of vedolizumab or infliximab. J Crohns Colitis*.* 2017;11(suppl 1):S318.

31. Allegretti JR, Barnes EL, Stevens B, et al. Predictors of clinical response and remission at 1 year among a multicenter cohort of patients with inflammatory bowel disease treated with vedolizumab. Dig Dis Sci*.* 2017;62(6):1590–6.

32. Amiot A, Grimaud JC, Peyrin-Biroulet L, et al. Effectiveness and safety of vedolizumab induction therapy for patients with inflammatory bowel disease. Clin Gastroenterol Hepatol*.* 2016;14(11):1593–1601.

33. Bhayat F, Blake A, Travis S. P668. Post-marketing experience of vedolizumab in inflammatory bowel disease: analysis of pneumonia and other respiratory tract infections. J Crohns Colitis*.* 2017;11(suppl 1):S421–2.

34. Bownik H, Grace R, Scott F, et al. Vedolizumab: efficacy and safety in patients with IBD – real world experience in a large academic university medical center. Am J Gastroenterol*.* 2015;110(suppl 1):S820.

35. Buer L, Høivik ML, Medhus AW, et al. P514. Combination treatment with vedolizumab and anti-TNF-α in inflammatory bowel disease: safety data. J Crohns Colitis. 2017;11(suppl 1):S341–2.

36. Chaparro M, Sierra-Ausin M, Mesonero F, et al. Effectiveness and safety of vedolizumab for the induction of remission in inflammatory bowel disease patients. United European Gastroenterol J*.* 2016;4(5 suppl):A264.

37. Chaudrey K, Whitehead D, Dulai PS, et al. Safety of vedolizumab in inflammatory bowel disease in a multi-center real world consortium. Gastroenterology*.* 2016;150(4 suppl 1):S974.

38. Christensen B, Rubin DT, Goeppinger S, et al. Endoscopic and histologic response and remission in inflammatory bowel disease patients initiating vedolizumab. Am J Gastroenterol*.* 2015;110(suppl 1):S783–4.

39. Christensen B, Micic D, Gibson PR, et al. P410. Vedolizumab is safe and effective for IBD, but has no effect on liver biochemistry in patients with concurrent PSC. J Crohns Colitis*.* 2017;11(suppl 1):S285–6.

40. Christopher B, Aoko O, Anderson E, et al. Clinical experience with vedolizumab in anti-TNF refractory IBD patients. United European Gastroenterol J*.* 2016;4(5 suppl):A465.

41. Crowell KT, Tinsley A, Williams ED, et al. PD7. Vedolizumab as rescue therapy in Crohn’s disease: results from a tertiary care center. Presented at: Annual Scientific Meeting of the American Society of Colon and Rectal Surgeons; April 30–May 4, 2016; Los Angeles, CA.

42. Drvarov O, AbuHashem R, Schunk N, et al. Skin and joint side effects in a subpopulation of anti-TNF experienced IBD patients, who respond to a treatment with vedolizumab, a humanized a4b7 integrin antibody. Gastroenterology*.* 2015;148(4 suppl 1):S865.

43. Dulai PS, Singh S, Narula N, et al. Vedolizumab for moderate to severely active inflammatory bowel disease: a multi-center U.S. consortium. Am J Gastroenterol. 2015;110(suppl 1):S809–10.

44. Eksteen B, Heatherington J, Oshiomogho JI, et al. Efficacy and safety of induction dosing of vedolizumab for reducing biliary inflammation in primary sclerosing cholangitis (PSC) in individuals with inflammatory bowel disease. Gastroenterology. 2016;150(4 suppl 1):S1268.

45. Ehehalt R, Schubert S, Stein D, et al. Treatment patterns of vedolizumab and anti-TNF-α use among patients with UC and CD in Germany: a multicenter retrospective chart review. Presented at: Advances in Inflammatory Bowel Diseases (AIBD) Annual Conference. December 8–10, 2016; Orlando, FL.

46. Gabriëls RY, de Graaf APJ, Meijssen MAC, et al. In a Dutch real-life inflammatory bowel disease score cohort with 90% prior anti-tumour necrosis factor failure, vedolizumab showed a 25% remission rate: a retrospective multicentre study. J Crohns Colitis*.* 2016;10(suppl 1):S416.

47. Gils A, Dreesen E, Compernolle G, et al. OP020. Recent anti-TNF exposure predicts lower vedolizumab trough concentrations in patients with Crohn's disease. J Crohns Colitis*.* 2017;11(suppl 1):S12.

48. Grace R, Bownik H, Scott F, et al. Infectious complications in IBD patients on immunomodulators, corticosteroids, and vedolizumab: is older age a predictor of higher complication rates or worsened response? Am J Gastroenterol*.* 2015;110(suppl 1):S823.

49. Gudsoorkar V, Chaikriangkrai K, Abraham B. Vitamin D deficiency is associated with persistent CRP elevation and a lower clinical response to vedolizumab treatment in Crohn’s disease patients. Am J Gastroenterol*.* 2015;110(suppl 1):S844–5.

50. Kaimakliotis P, Lazarev M, Bayless T, et al. Side effects related to vedolizumab use in clinical practice. Inflamm Bowel Dis*.* 2017;23(suppl 1):S54.

51. Kamperidis N, Cavazza A, Wahed M, et al. P391. Differential use of vedolizumab in ulcerative colitis and Crohn's disease. Real life results from 2 tertiary referral centres in the United Kingdom. J Crohns Colitis*.* 2017;11(suppl 1):S276–7.

52. Kassim O, Micic D, Christensen B, et al. P454. Can calcineurin inhibitors induce a durable remission that is maintained with vedolizumab in IBD? J Crohns Colitis. 2017;11(suppl 1):S309.

53. Khalid JM, Raluy-Callado M, Li Q, et al. P1897. Patterns of dose escalation amongst patients with ulcerative colitis and Crohn’s disease treated with vedolizumab vs. infliximab in the US. Presented at: United European Gastroenterology/American College of Gastroenterology Annual Scientific Meeting. October 14–19, 2016; Las Vegas, NV.

54. Kochhar G, Parikh M, Chaudrey K, et al. Mucosal healing with vedolizumab in ulcerative colitis and Crohn's disease: outcomes from the VICTORY Consortium. Inflamm Bowel Dis*.* 2017;23(suppl 1):S6.

55. Koh S, Zaghiyan K, Fleshner P. Safety and efficacy of the perioperative use of vedolizumab in medically refractory IBD patients. Does "gut-specificity" impact surgical morbidity? Dis Colon Rectum*.* 2016;59(5):e96–7.

56. Koliani-Pace JL, Chaudrey K, Kochhar G, et al. Rates and predictors of surgery in inflammatory bowel disease patients initiating vedolizumab therapy: results from the Victory Consortium. Gastroenterology*.* 2017;152(5 suppl 1):S779.

57. Kopylov U, Ron Y, Avni-Biron I, et al. Efficacy and safety of vedolizumab for induction of remission in inflammatory bowel disease – the Israeli experience. United European Gastroenterol J*.* 2016;4(5 suppl):A449–50.

58. Kopylov U, Sebastian S, Ron Y, et al. P366. The efficacy of vedolizumab for induction of clinical response and remission in anti-TNF naïve patients with inflammatory bowel disease – a multicenter European real world experience. J Crohns Colitis. 2017;11(suppl 1):S264–5.

59. Lenti MV, Johnston A, O'Connor A, et al. P227. Outcomes of anti-TNF versus vedolizumab therapy for ulcerative colitis: the Leeds experience. J Crohns Colitis. 2017;11(suppl 1):S192–3.

60. Lightner AL, Raffals LE, Mathis KL, et al. Postoperative outcomes in vedolizumab-treated patients undergoing abdominal operations for inflammatory bowel disease. J Crohns Colitis*.* 2017;11(2):185–90.

61. Lightner A, Mathis K, Sang Tse C, et al. P357. A multi-institutional report of postoperative outcomes in vedolizumab-treated patients undergoing major abdominal operations for inflammatory bowel disease. J Crohns Colitis*.* 2017;11(suppl 1):S258–9.

62. Lucci MB, Collins E, Cao B, et al. Sa1198. Initial vedolizumab cohort: patient characteristics and clinical response. Gastroenterology*.* 2015;148(4 suppl 1):S255.

63. Mahadevan U, Vermeire S, Lasch K, et al. Vedolizumab exposure in pregnancy: outcomes from clinical studies in inflammatory bowel disease. Aliment Pharmacol Ther. 2017;45(7):941–50.

64. Menon S, Makanyanga J, Mitchell T, et al. Results of vedolizumab use in the ‘real world.’ Presented at: Australian Gastroenterology Week. 10–12 October 2016; Adelaide, South Australia.

65. Meserve JD, Dulai P, Park S, et al. Vedolizumab does not increase the risk of enteric infections as compared to infliximab: a retrospective cohort study. Gastroenterology. 2017;152(5 suppl 1):S109–10.

66. Morganstern B, Singh N, Targan S, et al. Single-center experience of vedolizumab in patients with inflammatory bowel disease: Does age matter? Gastroenterology. 2015;148(4 suppl 1):S250.

67. Navaneethan U, Kommaraju KK, Edminster T, et al. Efficacy and safety of vedolizumab in elderly patients with inflammatory bowel disease. Gastroenterology. 2016;150(4 suppl 1):S812.

68. Navaneethan U, Edminister T, Zhu X, et al. Vedolizumab is safe and effective in elderly patients with inflammatory bowel disease. Inflamm Bowel Dis*.* 2017;23(4):E17.

69. Oppenheim S, Wasan SK, Noronha A, et al. Tu1371. A retrospective review of response rates and risk of complications among inflammatory bowel diseases patients treated with vedolizumab. Gastroenterology*.* 2015;148(4 suppl 1):S872.

70. Papamichail K, Rivals O, Billiet T, et al. Long-term outcome of IBD patients with primary non-response to anti-TNF therapy. J Crohns Colitis*.* 2015;9(suppl 1):S58–9.

71. Patel RJ, Grimes I. Vedolizumab in inflammatory bowel disease: a retrospective review of clinical efficacy, extra-intestinal manifestations and adverse reactions. Am J Gastroenterol*.* 2016;111(suppl 1):S317–8.

72. Peerani F, Narula N, Dulai PS, et al. Efficacy and predictors of outcomes of vedolizumab for ulcerative colitis in clinical practice. Gastroenterology*.* 2016;150(4 suppl 1):S392–3.

73. Plevris N, Manship TA, Deekae A, et al. P416. Real world data on the effectiveness and safety of vedolizumab in the treatment of Crohn's disease and ulcerative colitis: the Edinburgh experience. J Crohns Colitis*.* 2017;11(suppl 1):S288–9.

74. Raluy-Callado M, Alam N, Donaldson R, et al. A real-world study of outcomes in biologic-naïve patients with Crohn’s disease and ulcerative colitis initiating vedolizumab. J Crohns Colitis*.* 2016;10(suppl 1):S238.

75. Raluy-Callado M, Alam N, Wang R, et al. Hospitalisations and treatment discontinuation among patients with ulcerative colitis and Crohn’s disease treated with vedolizumab compared with infliximab. United European Gastroenterol J*.* 2016;4(5 suppl):A634.

76. Reynolds M, Raluy-Callado M, O'Hara D, et al. P-025. Hospitalizations and characteristics of patients with ulcerative colitis and Crohn's disease treated with vedolizumab in the United States. Inflamm Bowel Dis*.* 2016;22(suppl 1):S17.

77. Reynolds M, Alam N, Raluy M, et al. Hospitalisations and characteristics of patients with ulcerative colitis and Crohn’s disease treated with vedolizumab in real-world clinical practice: results from a multicentre study. J Crohns Colitis*.* 2016;10(suppl 1):S214.

78. Robinson K, Marshall L, Wright A, et al. N805. Do we need to monitor patients after vedolizumab infusion? J Crohns Colitis*.* 2017;11(suppl 1):S493–4.

79. Schils N, De Buck van Overstraeten A, Vermeire S, et al. P445. Perioperative use of vedolizumab seems not associated with short-term postoperative infectious complications in patients with Crohn's disease undergoing right hemicolectomy with ileocolonic anastomosis. J Crohns Colitis*.* 2017;11(suppl 1):S304.

80. Schmidt C, Langbein C, Atreya R, et al. P326. Efficacy of vedolizumab on patient-reported outcomes in ulcerative colitis patients: results from a prospective German observational study. J Crohns Colitis*.* 2017;11(suppl 1):S242–3.

81. Shivashankar R, Mendoza Ladd AH, Grace R, et al. Safety of vedolizumab use in patients with inflammatory bowel disease. Gastroenterology*.* 2017;152(5 suppl 1):S585.

82. Stevens BW, Shelton E, Sauk J, et al. Efficacy of vedolizumab as induction therapy in refractory IBD patients following multiple anti-TNF therapy failures. Gastroenterology. 2015;148(4 suppl 1):S869.

83. Stringfield S, Parry L, Sandborn W, et al. Patients on vedolizumab have a high rate of postoperative complications. Dis Colon Rectum*.* 2016;59(5):e96.

84. Tadbiri S, Grimaud JC, Peyrin-Biroulet L, et al. DOP025. Efficacy of vedolizumab on extraintestinal manifestation in patients with inflammatory bowel disease: a post-hoc analysis of the OBSERV-IBD cohort from the GETAID. J Crohns Colitis*.* 2017;11(suppl 1):S42.

85. Trefois Q, Descamps O, Coche JC, et al. Efficacy of vedolizumab as induction therapy for inflammatory bowel disease in a 'real-life' study. Acta Clinica Belgica*.* 2016;71(suppl 1):S7. Abstract 0005.

86. Turkeltaub JA, Waljee AK, Govani SM, et al. Vedolizumab persistence and tolerability in special populations is similar to other IBD users. Gastroenterology*.* 2017;152(5 suppl 1):S406.

87. Wice M, Oppenheim S, Miller H, et al. Efficacy and safety of vedolizumab in patients with inflammatory bowel disease in a large tertiary medical center. Am J Gastroenterol. 2016;111(suppl 1):S270.

88. Williamson KD, Slevin S, Willberg C, et al. Clinical and translational outcomes in patients with primary sclerosing cholangitis and inflammatory bowel disease receiving vedolizumab. J Hepatol*.* 2017;66(1 suppl):S544–5.

89. Ylisaukko-oja T, Eberl A, Aaltonen J, et al. P515. Evaluation of treatment persistence of vedolizumab among Finnish inflammatory bowel disease patients in real-life clinical practice (FINVEDO). J Crohns Colitis*.* 2017;11(suppl 1):S342.
